# Combinatorial targeting of Hippo-STRIPAK and PARP elicits synthetic lethality in gastrointestinal cancers

**DOI:** 10.1172/JCI155468

**Published:** 2022-05-02

**Authors:** Liwei An, Zhifa Cao, Pingping Nie, Hui Zhang, Zhenzhu Tong, Fan Chen, Yang Tang, Yi Han, Wenjia Wang, Zhangting Zhao, Qingya Zhao, Yuqin Yang, Yuanzhi Xu, Gemin Fang, Lei Shi, Huixiong Xu, Haiqing Ma, Shi Jiao, Zhaocai Zhou

**Affiliations:** 1 Department of Medical Ultrasound, Shanghai Tenth People’s Hospital, Tongji University Cancer Center, Tongji University School of Medicine, Shanghai, China.; 2 State Key Laboratory of Cell Biology, Shanghai Institute of Biochemistry and Cell Biology, Center for Excellence in Molecular Cell Science, Chinese Academy of Sciences, University of Chinese Academy of Sciences, Shanghai, China.; 3 State Key Laboratory of Genetic Engineering, Zhongshan Hospital, School of Life Sciences, Fudan University, Shanghai, China.; 4 Department of Laboratory Animal Center, Shanghai General Hospital, Shanghai Jiao Tong University School of Medicine, Shanghai, China.; 5 Department of Stomatology, Shanghai Tenth People’s Hospital, Tongji University School of Medicine, Shanghai, China.; 6 Institutes of Physical Science and Information Technology, Anhui University, Hefei, China.; 7 Department of Biochemistry and Molecular Biology, Tianjin Medical University, Tianjin, China.; 8 Department of Ultrasound, Zhongshan Hospital, Fudan University, Shanghai, China.; 9 Department of Oncology, Guangdong Provincial People’s Hospital, Guangdong Academy of Medical Sciences, Guangzhou, China.; 10 Collaborative Innovation Center for Cancer Personalized Medicine, Nanjing Medical University, Nanjing, China.

**Keywords:** Cell Biology, Gastroenterology, Drug therapy, Gastric cancer, Molecular biology

## Abstract

The striatin-interacting phosphatase and kinase (STRIPAK) complexes integrate extracellular stimuli that result in intracellular activities. Previously, we discovered that STRIPAK is a key machinery responsible for loss of the Hippo tumor suppressor signal in cancer. Here, we identified the Hippo-STRIPAK complex as an essential player in the control of DNA double-stranded break (DSB) repair and genomic stability. Specifically, we found that the mammalian STE20-like protein kinases 1 and 2 (MST1/2), independent of classical Hippo signaling, directly phosphorylated zinc finger MYND type–containing 8 (ZMYND8) and hence resulted in the suppression of DNA repair in the nucleus. In response to genotoxic stress, the cyclic GMP-AMP synthase/stimulator of IFN genes (cGAS/STING) pathway was determined to relay nuclear DNA damage signals to the dynamic assembly of Hippo-STRIPAK via TANK-binding kinase 1–induced (TBK1-induced) structural stabilization of the suppressor of IKBKE 1– sarcolemma membrane–associated protein (SIKE1-SLMAP) arm. As such, we found that STRIPAK-mediated MST1/2 inactivation increased the DSB repair capacity of cancer cells and endowed these cells with resistance to radio- and chemotherapy and poly(ADP-ribose)polymerase (PARP) inhibition. Importantly, targeting the STRIPAK assembly with each of 3 distinct peptide inhibitors efficiently recovered the kinase activity of MST1/2 to suppress DNA repair and resensitize cancer cells to PARP inhibitors in both animal- and patient-derived tumor models. Overall, our findings not only uncover what we believe to be a previously unrecognized role for STRIPAK in modulating DSB repair but also provide translational implications of cotargeting STRIPAK and PARP for a new type of synthetic lethality anticancer therapy.

## Graphical abstract

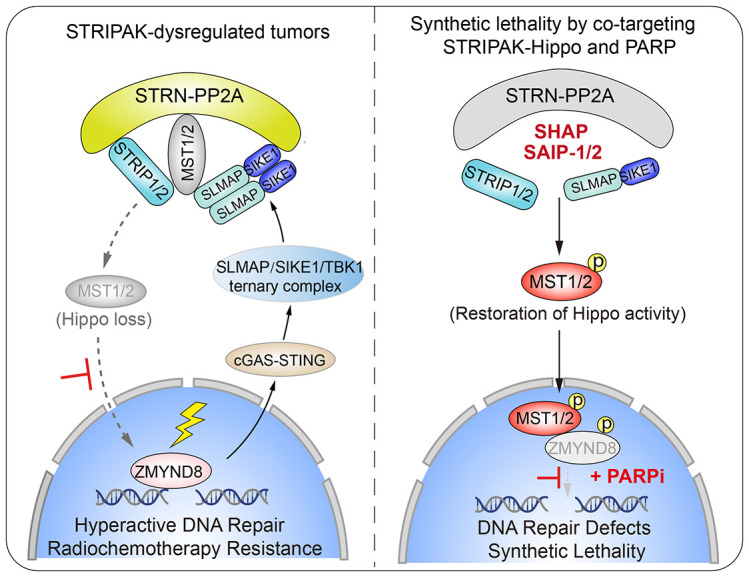

## Introduction

DNA double-stranded breaks (DSBs) constitute the most deleterious threat to genomic integrity, and the repair of DNA is essential for normal cell growth and animal development (1). Altered DNA repair capacity can be exploited by cancer cells to acquire selective growth advantages such as resistance to radiotherapy or chemotherapy (2). However, resulting differences between such repair in normal and tumor cells can make tumor cells vulnerable to targeted therapies. One such successful paradigm is the induction of synthetic lethality in homologous recombination–deficient (HR-deficient) tumors, which is achieved by treating the tumors with poly(ADP-ribose)polymerase inhibitors (PARPi) (3). Nevertheless, considering that only less than 20% of tumors exhibit intrinsic defects in DNA repair machinery, and nearly 90% of PARPi-responsive tumors eventually develop drug resistance (4), there is an urgent need to expand PARPi applications as well as to overcome the acquired drug resistance.

Striatin-interacting phosphatase and kinase (STRIPAK) complexes are supramolecular complexes conserved between various organisms. The centers of these complexes are formed each by the trimeric serine-threonine holoenzyme PP2A, which consists of scaffold subunit A (PP2AA), the catalytic subunit C (PP2AC), and striatin regulatory subunits (STRN1/3/4) (5, 6). The kinases incorporated into human STRIPAK complexes include mammalian STE20-like protein kinases 1 and 2 (MST1/2, the mammalian counterpart of the Hippo kinase), MST3/4, serine/threonine kinase 25 (STK25), misshapen-like kinase 1 (MINK1), and mitogen-activated protein kinase kinase kinase kinase 4 (MAP4K4) (5, 6). Additional scaffold components of STRIPAK complexes include striatin-interacting protein 1 and 2 (STRIP1/2), cerebral cavernous malformation 3 (CCM3) protein, suppressor of IKBKE 1 (SIKE1), and sarcolemma membrane–associated protein (SLMAP) and its paralog TRAF3-interacting protein 3 (TRAF3IP3) (7). Formation of an intact STRIPAK complex may not require all of these factors, but various combinations of these factors assemble to form different complexes that participate in diverse physiological functions such as tissue development and cellular homeostasis (8, 9).

Dysregulation of STRIPAK has been implicated in multiple human diseases and especially in cancer progression (10, 11). In this regard, we recently discovered an upregulation of STRN3 in gastric cancer (GC) cells, with STRN3 recruiting MST1/2 to the PP2A core enzyme to dephosphorylate MST1/2, a process that turns off the tumor-suppressive activity of Hippo signaling (12). By applying structure-guided peptide mimetics and chemical modification, we developed the stable and water-soluble peptide SHAP to recover the lost Hippo signal for cancer treatment. In addition, on the basis of the topological organization and (sub)structure of STRIPAK, we proposed a “2-arm” assembly model of the STRIPAK complexes (7). In this model, STRN3 acts as a central backbone to directly interact with 2 “molecular arms,” i.e., STRIP1/2 and SIKE1-SLMAP; these 2 arms further bind kinases in a phosphorylation-dependent manner to recruit them as substrates of PP2A (7). More recently, the cryo-electron microscopy (cryo-EM) structure of STRIPAK was determined and largely overlaps with the above assembly model, especially from the perspective of the STRIP1 arm, providing a further structural basis for understanding the functions of these complexes (13).

Recently, there has been increasing evidence showing that STRIPAK acts as a regulatory hub for orchestrating upstream signals to initiate Hippo signaling. Specifically, in response to extracellular pro-proliferative stimuli, STRIPAK can interact with and dephosphorylate the Hippo kinases MST1 and MST2 to release their suppressive effect on the downstream effectors YAP/TAZ, promoting cell proliferation and tissue regeneration (7, 14–18). However, it has not yet been determined whether STRIPAK complexes can sense intracellular stimuli such as genotoxic stress to regulate DNA damage responses.

In this study, we identified the Hippo-containing STRIPAK complex as a sensor and regulator of DSB repair and genomic stability. TANK-binding kinase 1 (TBK1), downstream of cyclic GMP-AMP synthase/stimulator of IFN genes (cGAS/STING) signaling, was found to respond to DNA damage by structurally stabilizing the SIKE1-SLMAP arm to facilitate a tightened assembly of the STRIPAK-Hippo complex, resulting at initial stages in an inactivation of MST1/2. Otherwise, MST1/2 were shown to directly phosphorylate zinc finger MYND-type containing 8 (ZMYND8) to suppress its recruitment to DSBs in the nucleus. As such, loss of the Hippo signal was determined to robustly stimulate DSB repair and endow cancer cells with resistance to radio- and chemotherapy. Accordingly, a negative correlation between Hippo activity and acquired resistance to PARPi was also revealed for GC. On the basis of these findings, we further developed a synthetic lethality therapy in which the STRIPAK assembly was targeted via a combination of approaches to recover the kinase activity of MST1/2 and therefore resensitize gastrointestinal tumors to the effects of PARPi.

## Results

### The Hippo-containing STRIPAK complex is essential for DSB repair.

**Figure 1:**
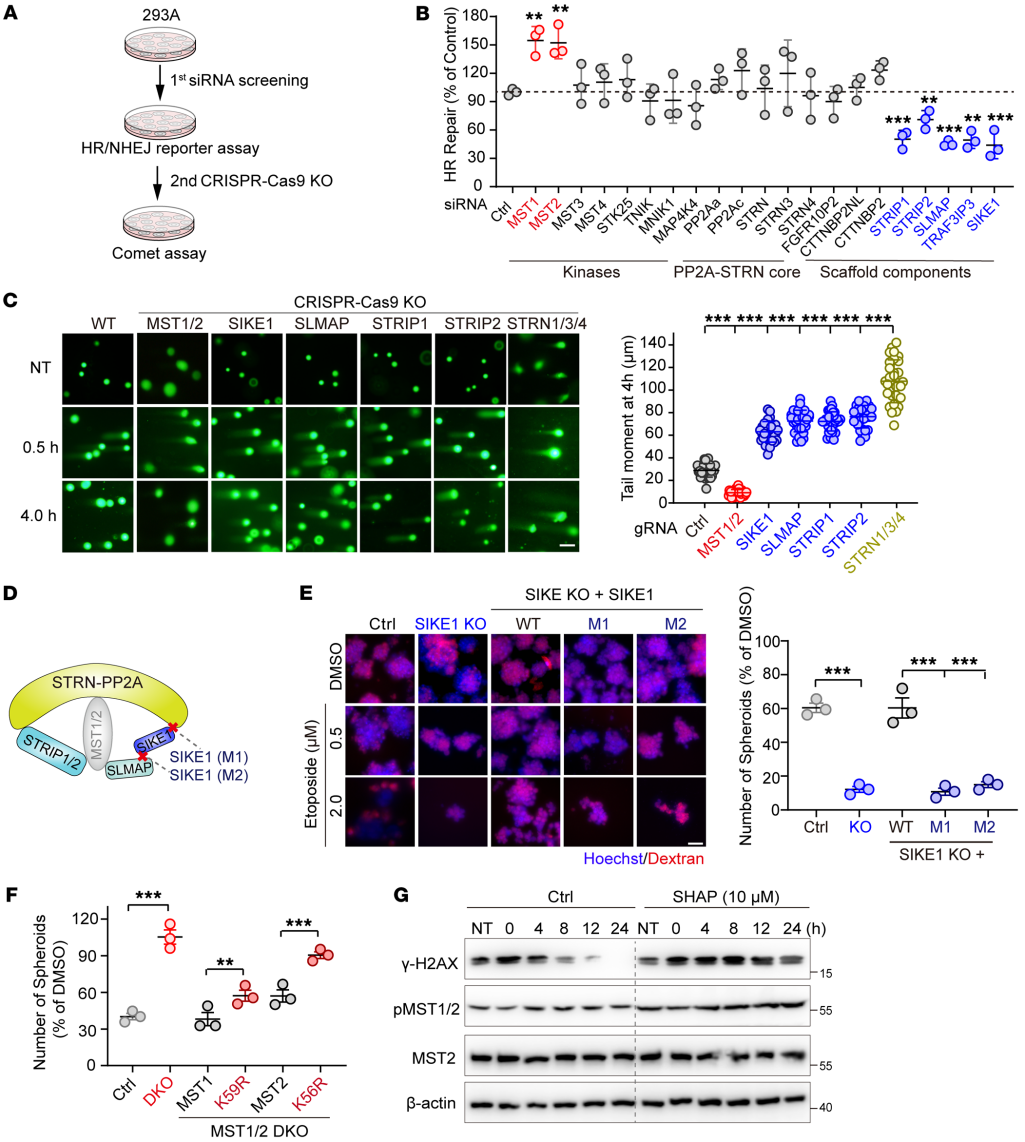
The Hippo-containing STRIPAK complex is essential for DSB repair. (**A**) Schematic illustration of the miniscreen of STRIPAK in DSB repair. (**B**) Plot showing the regulation of HR repair by the siRNA-mediated knockdown components of STRIPAK (*n =* 3). (**C**) Images showing the results of the indicated HGC-27 cells exposed to IR (10 Gy) and collected 0.5 and 4 hours after treatment before being subjected to a neutral comet assay (scale bar: 50 μm), and a plot providing quantifications of the tail moment at 4 hours (*n =* 30 cells/group). (**D**) Schematic presentation of the assembly of the STRIPAK-MST1/2 complex and SIKE1 mutants (M1 and M2). (**E**) Images and plot showing that SIKE1 promoted genomic stability within STRIPAK. HGC-27 cells (WT and its derivatives) were first treated with etoposide for 1 hour before being subjected to the sphere formation assay. Scale bar: 60 μm. (**F**) Plot showing that MST1/2 limited cancer cell sphere formation in a manner dependent on their kinase activity of MST1/2. HGC-27 cells (WT and its derivatives) were treated and processed as in **E**. (**G**) Gels showing that SHAP treatment attenuated DNA repair capacity. HGC-27 cells pretreated with etoposide for 1 hour were further incubated with or without SHAP peptides. **P < 0.01 and ***P < 0.001, by 1-way ANOVA with Dunnett’s post hoc test, compared with control (**B** and **C**) and unpaired Student *t* test (**E** and **F**). See also Supplemental Figures 1 and 2. Ctrl, control.

To determine whether STRIPAK complexes are involved in the cellular response to DNA damage, we performed an siRNA miniscreen targeting individual components of the complexes in 2 well-characterized DSB reporter systems, specifically to identify potential regulators (first step), and then performed a neutral comet assay to validate the roles of candidate hits in DNA repair (second step) (Figure 1A). Intriguingly, we found that depletion of the Hippo kinases MST1/2 greatly stimulated both HR and nonhomologous end-joining (NHEJ) repair capacity, whereas deficiency of STRIPAK scaffold components including SIKE1, STRIP1/2, SLMAP, or TRAF3IP3 dramatically attenuated such capacity (Figure 1B and Supplemental Figure 1A; supplemental material available online with this article; https://doi.org/10.1172/JCI155468DS1). Moreover, the inhibitory effects on DSB repair seemed to be specific for MST1/2 kinases, as knockdown of other kinase components of STRIPAK such as MST3, MST4, STK25, TRAF2 and NCK interacting kinase (TNIK), MNIK1, and MAP4K4 failed to achieve such inhibition (Figure 1B and Supplemental Figure 1A). To validate the primary screening results, we next generated individual KO cell lines against MST1/2, SIKE1, SLMAP, STRIP1, and STRIP2 (Supplemental Figure 1B) and reexamined the DSB repair efficiency levels of these cells. Consistent with observations in the siRNA-mediated knockdown approach, individual KOs of MST1 and MST2 each increased both HR and NHEJ repair efficiency levels to some extent, and the double-KO (MST1/2-DKO) did so to a greater extent (Supplemental Figure 1C). Conversely, KO of the indicated scaffold components significantly impaired the DSB repair capacity (Supplemental Figure 1C).

Next, we carried out a neutral comet validation assay, whose results similarly recapitulated the DSB reporter assay observations. That is, the tail moments were dramatically shorter in the MST1/2-DKO cells, and markedly longer in the scaffold component–KO cells, than in WT control cells 4 hours after irradiation (IR) treatment, although DNA damage was induced to comparable levels at 0.5 hours (Figure 1C). Interestingly, although siRNA-mediated knockdown of any single member of the STRN family (STRN1/3/4) did not alter DSB repair efficiency (Figure 1B and Supplemental Figure 1A), triple-KO cells (STRN-TKO) displayed significantly longer tail moments than did the WT group (Figure 1C and Supplemental Figure 1D), suggesting a functional redundancy of STRNs in response to DNA damage stimuli. Also, we monitored the IR-induced foci formation of DNA damage markers such as γ-H2AX, 53BP1, and RAD51, each at various time points, and the results of this monitoring revealed that the damaged DNA was repaired much more rapidly in MST1/2-deficient cells, and more slowly in SIKE1/SLMAP-depleted cells, than in parental WT cells (Supplemental Figure 1E).

The opposite roles of STRIPAK scaffold components and Hippo kinases in DNA repair prompted us to speculate that they may function as an intact complex — especially considering that STRIP1/2 and SIKE1-SLMAP, the 2 “molecular arms” of the STRIPAK complex essential for recruiting kinases (Figure 1D), showed profound effects in both reporter and neutral comet DNA repair assays (Figure 1, B and C). To validate this hypothesis, we first generated 2 mutants of SIKE1 shown to specifically disrupt its binding to either STRNs (M1) or SLMAP (M2) within the complex (ref. 7, Figure 1D, and Supplemental Figure 1F). We then performed a clonogenic survival assay (Supplemental Figure 1G) and a sphere formation assay (Figure 1E) for cells of the GC cell line HGC-27 subjected to genotoxic stress. We found that SIKE1-KO cells were more sensitive to etoposide treatment than were WT cells (Figure 1E and Supplemental Figure 1G). Notably, reconstitution of SIKE1-KO cells with WT SIKE1 restored cell viability and the sphere-forming ability to levels comparable to those of parental WT cells under both experimental settings, but reconstitution with the M1 or M2 mutant of SIKE1 failed to do so (Figure 1E and Supplemental Figure 1G), indicative of the ability of SIKE1 to promote DNA repair and genomic stability in the context of an intact STRIPAK. Similarly, we further validated the functional importance of STRIPAK integrity from the perspective of SLMAP by using mutants shown to disrupt its interaction with either SIKE1 (4LD) or MST1/2 (ΔFHA) (ref. 7 and Supplemental Figure 1H). Consistently, although SLMAP deficiency sensitized HGC-27 cells to etoposide, reintroduction of WT SLMAP, but not its mutants 4LD or ΔFHA, reversed the cell survival rate to parental WT levels (Supplemental Figure 1I).

Taken together, these results uncovered an essential role of the Hippo-containing STRIPAK complex in DSB repair control and genomic stability.

### MST1/2 suppress DSB repair in a fashion dependent on their kinase activities.

To validate the observed hyperactive DNA repair capacity of MST1/2-deficient cells, we compared the repair dynamics of WT and MST1/2-DKO cells after they were treated with either etoposide or IR and found that damaged DNA was repaired much more rapidly in MST1/2-DKO cells than in WT cells (Supplemental Figure 2, A and B). Next, to evaluate whether the kinase activity was required for the MST1/2-mediated inhibition of DSB repair, we stably reconstituted MST1/2-DKO cells with constructs encoding WT MST1, its kinase-inactive mutant K59R, as well as WT MST2 or its kinase-inactive mutant K56R (Supplemental Figure 2C), and compared their abilities to modulate HR repair and to form spheres in response to etoposide. Compared with WT cells, MST1/2-DKO cells consistently displayed a hyperactive DNA repair ability (Supplemental Figure 2D), and MST1/2 DKO strongly protected cancer cells from etoposide-induced cell death (Figure 1F and Supplemental Figure 2E). In sharp contrast, reconstitution of MST1 or MST2, but not of their corresponding inactive mutants K59R and K56R, reversed the elevated HR repair efficiency (Supplemental Figure 2D) and resensitized HGC-27 cells to etoposide treatment (Figure 1F and Supplemental Figure 2E). Importantly, treating 293A cells with the SHAP peptide, an agonist for MST1/2 kinases (12), markedly attenuated the repair dynamic process upon induction of DNA damage (Figure 1G), demonstrating that MST1/2 suppress DSB repair in a manner dependent on their kinase activities.

### The STRIPAK complex undergoes dynamic assembly upon DNA damage.

**Figure 2:**
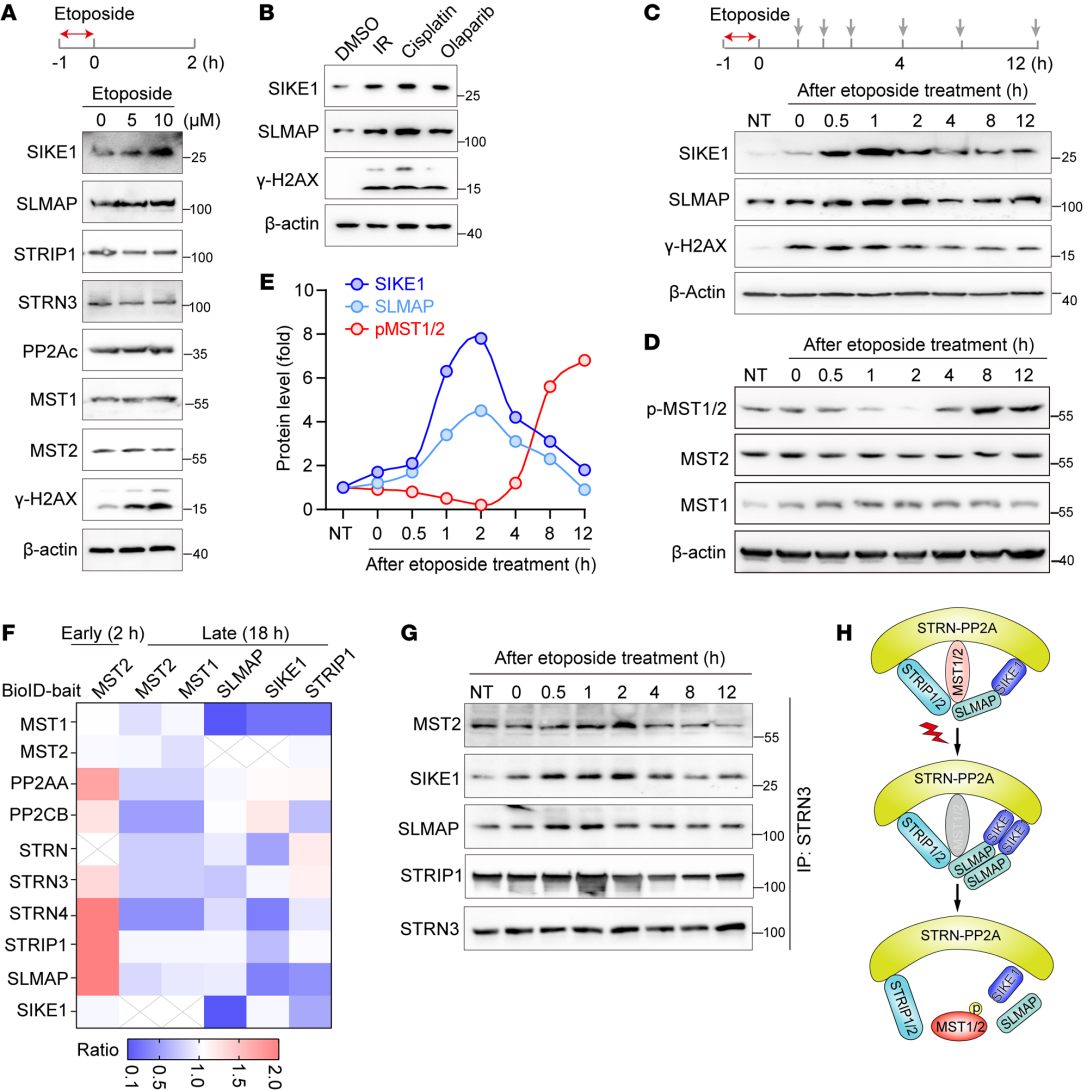
STRIPAK-MST1/2 undergoes dynamic assembly in response to DNA damage. (**A** and **B**) Gels showing DNA damage–triggered stabilization of the SIKE1-SLMAP arm at the protein level. Here, 293A cells were pretreated with the indicated dose of etoposide (**A**) or with other DNA-damaging agents (**B**) for 1 hour, and then harvested 2 hours after removal of the chemicals. (**C**–**E**) Gels and plots showing a DNA damage–induced mutually exclusive pattern between SIKE1-SLMAP protein levels and MST1/2 kinase activity. Cells were exposed to 10 μM etoposide for 1 hour and then collected at the indicated time points after removal of the drug to determine the (**C**) protein levels of both SIKE1 and SLMAP in 293A cells and (**D**) MST1/2 kinase activity in HGC-27 cells. (**E**) Quantitative data from HGC-27 cells. (**F**) Dynamic assembly of STRIPAK-MST1/2 induced by DNA damage. 293A cells transiently transfected with the indicated constructs for 24 hours were treated with DMSO or etoposide (1 h) and then harvested at an early (2 h) or late (18 h) stage before being subjected to BioID analysis. (**G**) Co-IP assay to validate the BioID results. 293A cells were treated and harvested as in **C** before processing for STRN3 IP. (**H**) Cartoon representation of a loosening of the assembly of the Hippo-containing STRIPAK complex in response to DNA damage. See also Supplemental Figure 3.

Previously, we and others have shown that STRIPAK complexes undergo dynamic assembly in response to environmental cues (7, 18). Given its role in DSB repair, we speculated that the Hippo-containing STRIPAK complex may (dis)assemble in response to genotoxic stress. To test this possibility, we first performed Western blotting to examine the levels of each of the STRIPAK component proteins. Surprisingly, we found that expression levels of SIKE1 and SLMAP, but not other subunits, were markedly increased in a dose- and time-dependent manner upon etoposide treatment (Figure 2A and Supplemental Figure 3A). Similarly, we also observed elevated protein levels of both SIKE1 and SLMAP after exposure of the cells to several other genotoxic agents including IR, cisplatin, and olaparib (Figure 2B and Supplemental Figure 3B).

To better monitor the dynamic changes in the levels of SIKE1 and SLMAP upon DNA damage, we first treated 293A cells with etoposide for 1 hour to induce robust DNA damage and further collected samples at various time points after removal of the chemical drug (Figure 2C). Interestingly, we found that both SIKE1 and SLMAP proteins were transiently upregulated at early time points but gradually returned to their respective basal levels at later stages, featuring a pattern highly similar to that shown by the DNA damage marker γ-H2AX (Figure 2C and Supplemental Figure 3C). We also observed accumulations of SIKE1 and SLMAP in HGC-27 cells (Supplemental Figure 3D). Consistent with these results, the kinase activity of MST1/2, as reflected by the levels of phosphorylated MST1 (Thr183)/MST2 (Thr180) (hereafter referred to as p-MST1/2), was attenuated in the early stages (~2 h) but robustly enhanced in the later stages after induction of DNA damage in both HGC-27 cells (Figure 2, D and E) and 293A cells (Supplemental Figure 3C).

Given the early-stage upregulation of SIKE1 and SLMAP protein levels and the late-stage activation of MST1/2, we speculated that the interaction of MST1/2 with the rest of the complex may become enhanced immediately after induction of DNA damage to relieve MST1/2-mediated suppression of DSB repair — but then later become decreased along with the progress of DSB repair to recover MST1/2 kinase activity. To test this hypothesis, we next carried out a proximity labeling–based mass spectrum (MS) assay (BioID) (19), specifically to characterize the dynamic assembly of the Hippo-containing STRIPAK complex (Supplemental Figure 3E). To this end, we first performed MST2 BioID at early (2 h) and late (18 h) time points after induction of DNA damage (Figure 2F). Interestingly, we indeed observed enhanced interactions between MST2 and STRIP1, SLMAP, and STRN3/4 at 2 hours, whereas the MST2-STRIPAK association was markedly decreased at 18 hours (Figure 2F). Conversely, the associations of MST1/2 with SLMAP, SIKE1, and STRIP1 were also dramatically reduced from the perspective of the SIKE1-SLMAP/STRIP1 interactome at 18 hours (Figure 2F). We next validated the BioID results by performing a co-IP assay following the same procedure as described in Figure 2C. The results indicated that the strengths of the associations of STRN3 with SIKE1, SLMAP, and MST2 were markedly increased at the early stages, but gradually decreased at the later stages in both 293A (Figure 2G and Supplemental Figure 3F) and HGC-27 cells (Supplemental Figure 3D).

On the basis of these results, we proposed a loosening of the assembly of the Hippo-containing STRIPAK complex in response to DNA damage (Figure 2H).

### The cGAS-STING sensor relays DNA damage signal to the STRIPAK dynamic assembly.

**Figure 3:**
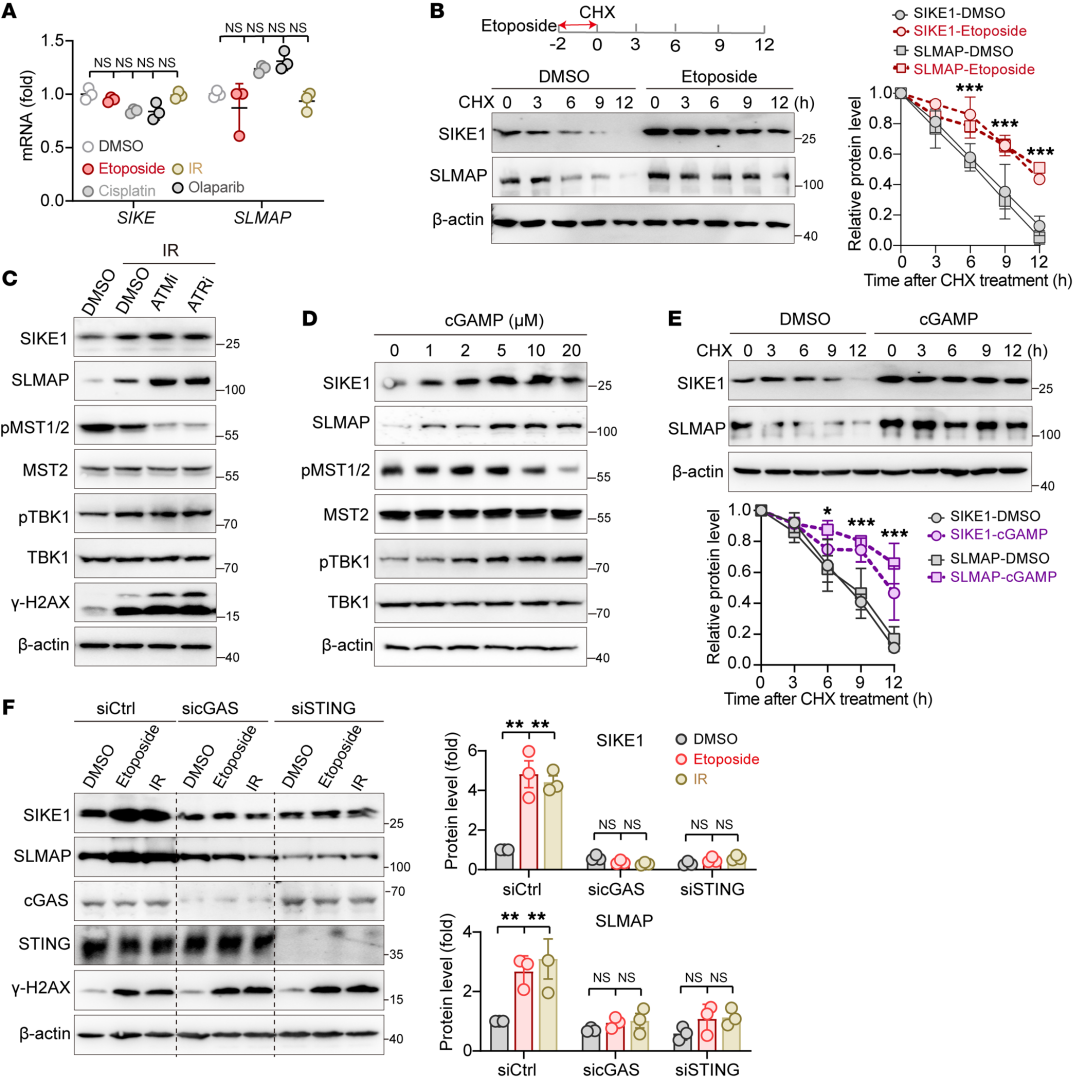
The cGAS-STING sensor relays the DNA damage signal to the STRIPAK assembly. (**A**) Lack of effect of DNA damage on *SIKE1* and *SLMAP* transcription (*n =* 3). (**B**) DNA damage–induced stabilization of SIKE1 and SLMAP proteins. 293A cells pretreated with etoposide for 2 hours were analyzed using a CHX assay. (**C** and **D**) Correlation of the activation of the cGAS/STING pathway with SIKE1 and SLMAP protein accumulation and MST1/2 inactivation. 293A cells treated with DMSO, KU-55933 (10 μM, ATMi), or VE-821 (10 μM, ATRi) were subjected to (**C**) IR treatment, or (**D**) were simply treated with the indicated dose of cGAMP for 2 hours. Cells were harvested 2 hours after treatment. (**E**) cGAS-STING activation–induced stabilization of SIKE1 and SLMAP at the protein level. 293A cells pretreated with 5 μM cGAMP for 2 hours were analyzed using the CHX assay. (**F**) Gels showing that depletion of cGAS or STING blocked DNA damage–induced stabilization of SIKE1 and SLMAP. 293A cells transfected with siRNAs against cGAS or STING were subjected to IR or etoposide treatment and were collected 2 hours after treatment (*n =* 3). *P < 0.05, **P < 0.01, and ***P < 0.001, by 1 way ANOVA with Dunnett’s post hoc test (**A** and **F**) and unpaired Student *t* test (**B** and **E**). See also Supplemental Figure 4.

To investigate how the nuclear DNA damage signal is transduced to and interpreted as the dynamic assembly of the STRIPAK complex, we monitored the variations of SIKE1 and SLMAP expression levels in response to genotoxic stress. First, we were able to exclude the possibility that the increased protein levels were caused by elevated transcriptional activity, as mRNA levels of both *SIKE1* and *SLMAP* in cells treated with diverse DNA-damaging agents were comparable to those in the untreated control (Figure 3A). Next, we performed a cycloheximide (CHX) assay to monitor the protein turnover rates of SIKE1 and SLMAP upon induction of DNA damage. In sharp contrast to the rapid degradations of SIKE1 and SLMAP proteins in the control group treated with DMSO, cells treated with etoposide showed much slower turnover rates of SIKE1 and SLMAP proteins (Figure 3B), indicating that both proteins were stabilized and accumulated upon the occurrence of DNA damage.

Regarding the upstream machinery regulating the expression levels of SIKE1 and SLMAP, we speculated that the cytosolic DNA sensor cGAS and the downstream effector STING pathway have spatiotemporal potency in relaying nuclear DNA damage signals to the dynamic assembly of STRIPAK (6, 20). To test this hypothesis, we examined the potential effects of ataxia telangiectasia-mutated (ATM) or ataxia telangiectasia and rad3-related (ATR) on the fluctuation of SIKE1 and SLMAP protein levels — with the rationale that inhibition of ATM or ATR would boost DNA damage–induced activation of cGAS/STING signaling (20) and hence induce an accumulation of SIKE1 and SLMAP proteins. We reproducibly detected the accumulation of SIKE1 and SLMAP proteins along with elevated TBK1 phosphorylation after IR. Treatment with KU-55933 (ATMi) or VE-821 (ATRi) not only augmented γ-H2AX and TBK1 phosphorylation, but also further increased the levels of SIKE1 and SLMAP proteins (Figure 3C). Accompanying the accumulation of SIKE1 and SLMAP proteins was a marked reduction in p-MST1/2 levels 2 hours after IR (Figure 3C).

The above results suggested a positive correlation between activation of the cGAS/STING pathway (TBK1 phosphorylation) and STRIPAK assembly (SIKE1-SLMAP upregulation). To confirm this correlation, we next examined whether cGAMP, an agonist of cGAS, could mimic such an effect on STRIPAK. Similar to the results for the etoposide treatment, we indeed observed a time- and dose-dependent stimulatory pattern of SIKE1 and SLMAP protein accumulations upon treatment of cells with cGAMP (Figure 3D and Supplemental Figure 4A). Consistent with the results obtained in the setting of DNA damage, increased TBK1 phosphorylation and decreased p-MST1/2 levels were also recapitulated by the cGAMP treatment (Figure 3D). Accordingly, cGAMP treatment similarly stabilized both SIKE1 and SLMAP protein levels (Figure 3E). To further evaluate the role of the cGAS/STING pathway in mediating DNA damage–induced SIKE1 and SLMAP stabilization, we individually depleted cGAS or STING and reexamined their protein levels upon effecting DNA damage. Strikingly, although SIKE1 and SLMAP protein levels were markedly increased in WT cells after they were subjected either to an etoposide or IR treatment, depletion of either cGAS or STING entirely blocked such accumulation of these 2 proteins in both 293A (Figure 3F) and HGC-27 cells (Supplemental Figure 4B). We also observed similar results when we treated 293A cells with inhibitors against cGAS (RU.521) or STING (c-176) (Supplemental Figure 4C).

### Activated TBK1 stabilizes SIKE1-SLMAP by forming a ternary complex.

**Figure 4:**
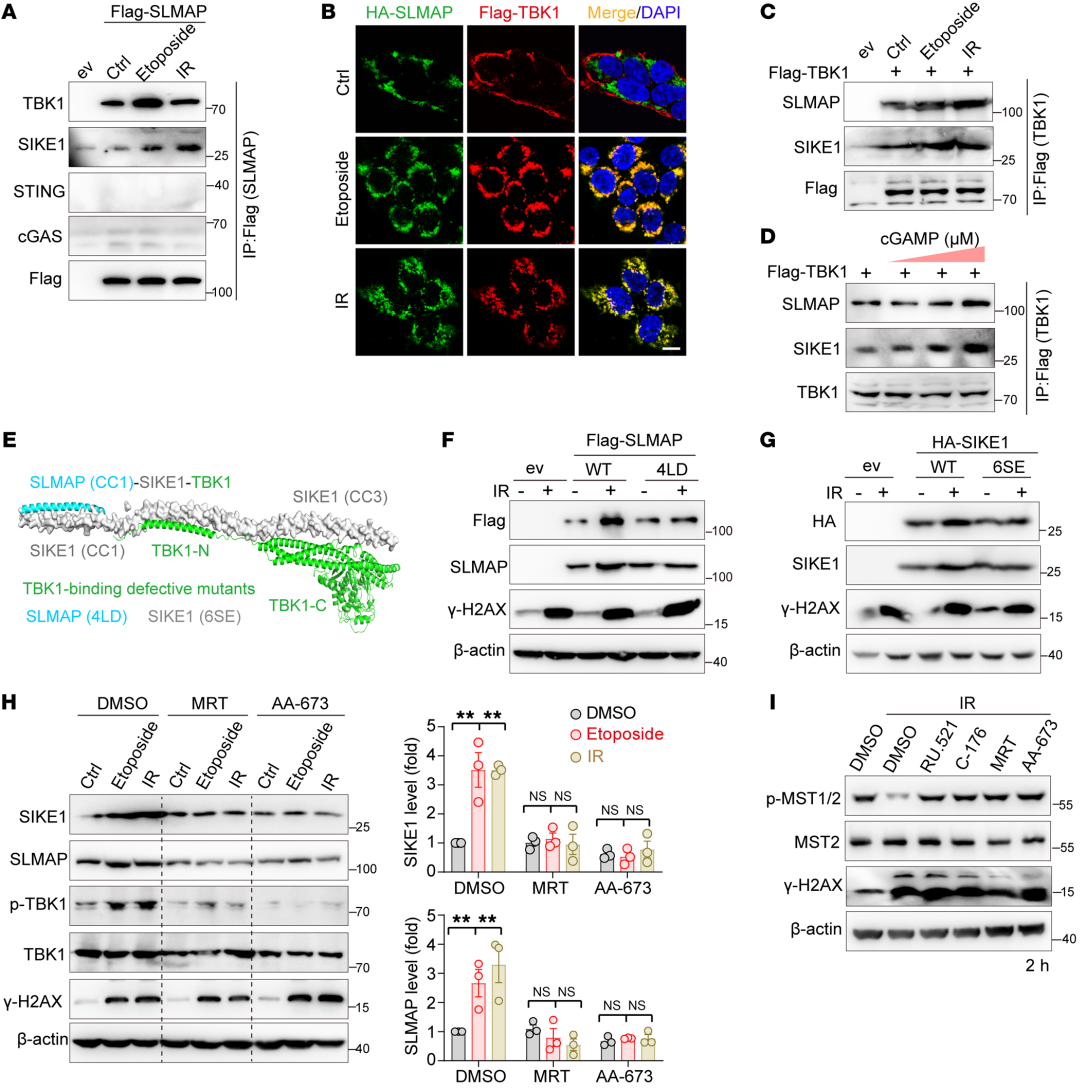
Activated TBK1 stabilizes SIKE1 and SLMAP proteins. (**A**–**C**) Gels and images showing that DNA damage triggered an enhancement of SLMAP-TBK1 interactions. Samples of 293FT cells were (**A**) transiently transfected with Flag-SLMAP, (**B**) cotransfected with Flag-TBK1 and HA-SLMAP, or (**C**) transfected with Flag-TBK1. Cells were treated as indicated and harvested 2 hours after treatment for either the (**A** and **C**) co-IP or (**B**) immunofluorescence assay. Scale bar: 10 μm. (**D**) Gels showing that cGAS-STING activation enhanced TBK1-SIKE1-SLMAP ternary interactions. 293FT cells transfected with Flag-TBK1 were treated with cGAMP for 1 hour before being processed for an IP assay conducted 2 hours after the treatment. (**E**) Hypothetical structural model of the SLMAP-SIKE1-TBK1 ternary complex (PDB: 6AKM and TBK1 from the AlphaFold database). TBK1-binding–defective mutants of SLMAP and SIKE1 are shown. (**F** and **G**) Gels showing that accumulation of SIKE1 and SLMAP required their interaction with TBK1. (**F**) HGC-27 SLMAP-KO cells or (**G**) SIKE1-KO cells were reconstituted with either WT or TBK1-binding–defective mutants and treated with 10 Gy IR before being harvested 2 hours after treatment. (**H**) Gel and plots showing that TBK1 inhibition attenuated DNA damage–induced stabilization of SIKE1 and SLMAP proteins. 293A cells pretreated with MRT (10 μM) or AA-673 (50 μM) were subjected to IR or etoposide treatment as described in **F**. *n =* 3. **P < 0.0, by 1-way ANOVA with Dunnett’s post hoc test, compared with DMSO. (**I**) Gel showing that inhibition of the cGAS/STING/TBK1 pathway failed to inactivate MST1/2 at an early stage after DNA damage. HGC-27 cells were treated with the indicated inhibitors and processed as in **H**. See also Supplemental Figure 4. ev, empty vehicle.

Seeking the potential mechanism or mechanisms through which cGAS/STING signaling modulates SIKE1-SLMAP protein turnover, we next examined whether SIKE1 or SLMAP may physically interact with core components of this pathway upon the occurrence of DNA damage. To this end, we induced the expression of Flag-tagged SLMAP in 293A cells and performed a co-IP assay following treatment with IR or etoposide. Interestingly, this analysis revealed a specific interaction of SLMAP with TBK1 but not with cGAS or STING, and this interaction with TBK1 was further enhanced after the induction of DNA damage (Figure 4A and Supplemental Figure 4D). Accordingly, the SLMAP and TBK1 proteins exhibited strong colocalization upon the induction of DNA damage (Figure 4B). Since SIKE1 has been well characterized as a TBK1 binder and substrate (21, 22), and since SIKE1 was observed to interact with SLMAP in a crystal structure (7), it is likely that these 3 factors form a ternary complex upon the occurrence of DNA damage. Supporting this notion, a reciprocal Flag-TBK1 co-IP assay revealed that the interaction of TBK1 with both SIKE1 and SLMAP was enhanced in response to either DNA damage (Figure 4C and Supplemental Figure 4E) or cGAMP treatment (Figure 4D and Supplemental Figure 4F).

SIKE1 has been shown to directly bind the coil-coiled domain of SLMAP (residues 161–230) (7) and the C-terminal region of TBK1 (residues 601–729; ref. 21) via its N- and C-termini, respectively, hinting that SIKE1 may act as a molecular linker to mediate the interaction between SLMAP and TBK1. Indeed, an in vitro cell-free assay using purified SLMAP and TBK1 failed to detect a direct interaction between these 2 proteins (Supplemental Figure 4G). Moreover, a further co-IP assay demonstrated that the 4LD mutant of SLMAP (SIKE1-binding defective) also failed to interact with TBK1 (Supplemental Figure 4H). Combining the previous structural information (Protein Data Bank [PDB]: 6AKM and AlphaFold Protein Structure Database), we thus proposed a structural model in which a SIKE1-SLMAP heterodimer binds to the C-terminal coil-coiled domain of TBK1 (Figure 4E). Note that mutation of 6 serine residues, specifically those clustered in the C-terminal CC3 portion of SIKE1, to glutamate (the 6SE mutant) was previously shown to disrupt its binding of SIKE1 with TBK1 (22) and was used here to generate a TBK1-binding–defective mutant (Figure 4E).

Next, we examined whether TBK1 activation and association with SIKE1 and SLMAP would be required for DNA damage–induced stabilization of SIKE1 and SLMAP proteins. In this regard, we reproducibly observed a dramatic increase in the protein levels of WT SLMAP and SIKE1 after IR; however, hardly any change in their protein levels was detected when using the TBK1-binding–defective 4LD mutant of SLMAP (Figure 4F and Supplemental Figure 4I) and the 6SE mutant of SIKE1 (Figure 4G). Furthermore, because of the phosphorylation dependence of the TBK1-SIKE1 interaction, treatment of TBK1 with inhibitors such as MRT or AA-673 not only greatly attenuated its association with the SIKE1-SLMAP heterodimer (Supplemental Figure 4J), but also efficiently blocked SIKE1 and SLMAP protein accumulation (Figure 4H). More important, such treatment failed to inactivate the kinase activity of MST1/2 two hours after DNA damage, findings consistent with the results obtained from targeting the cGAS-STING sensors (Figure 4I and Supplemental Figure 4K). Collectively, these results demonstrated that activated TBK1 formed a complex with and stabilized the SIKE1-SLMAP arm of STRIPAK, facilitating a tightened STRIPAK-MST1/2 complex.

### DNA damage triggers nuclear localization of MST1/2 to limit DNA repair capacity.

Realizing the importance of the Hippo-containing STRIPAK complex in sensing DNA damage, we next moved on to dissect the mechanism through which MST1/2 suppress DNA repair activity. Considering that cytoplasmic MST2 has been reported to act downstream of ATM/RASSFIA signaling to promote apoptosis via the LATS/YAP/p73 axis in response to IR or chemotherapeutic drugs (23–27) and that activated YAP/TAZ was also shown to regulate DSB repair and contribute to acquired drug resistance (28, 29), we examined whether MST1/2 regulate DNA repair via the classical LATS1/2-YAP/TAZ signaling cascade. Surprisingly, in sharp contrast to MST1/2 depletion, we found that knockdown of LATS1/2 did not noticeably affect HR or NHEJ repair capacity (Supplemental Figure 5, A–C). Meanwhile, the efficiency of DSB repair was only mildly reduced upon codepletion of YAP and TAZ (Supplemental Figure 5, A–C). In addition, the transcription levels of YAP/p73-responsive genes, such as *PUMA* (23, 30), *BAX*, and *BCL2* (31), and YAP/TEAD-responsive genes, such as *CTGF* and *CYR61* (32), were not noticeably altered 12 hours after treatment with various DNA-damaging agents (Supplemental Figure 5, D and E).

**Figure 5:**
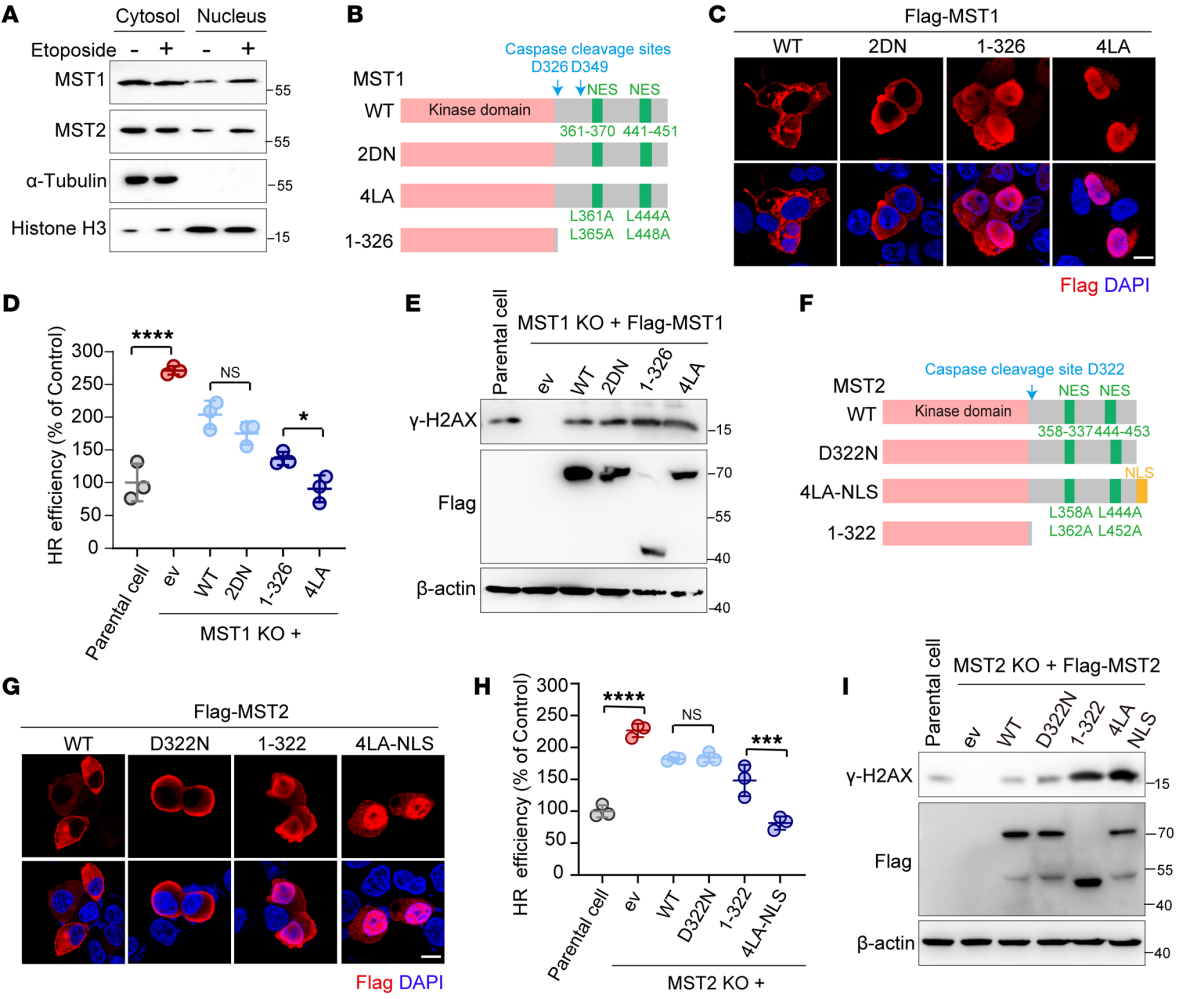
MST1/2 kinases suppress DSB repair in the nucleus. (**A**) Gels showing that DNA damage enhanced nuclear localization of MST1/2 kinases. Samples of 293A cells treated with or without etoposide were subjected to the respective cell fractionation assays. (**B** and **C**) Schematic presentation of MST1 constructs (WT and mutants) (**B**) and their subcellular localization patterns (**C**). Scale bar: 10 μm. (**D** and **E**) Plot and gel showing that MST1 inhibited DSB repair in a manner dependent on its nuclear localization. 293A cells (WT and MST1-KO and its derivatives) were subjected to either (**D**, *n =* 3) a HR reporter assay or (**E**) Western blot analysis of γ-H2AX levels in HGC-27 MST1-KO cells expressing the indicated MST1 mutation 12 hours after IR treatment. (**F** and **G**) Schematic presentation of MST2 constructs (WT and mutants) (**F**) and their subcellular localization (**G**). Scale bar: 10 μm. (**H** and **I**) Plot and gel showing that MST2 inhibited DSB repair in a manner dependent on its nuclear localization. (**H**, *n =* 3) 293A MST2-KO cells and (**I**) HGC-27 MST2-KO cells were treated and processed as in **D** and **E**. *P < 0.05, ***P < 0.001, and ****P < 0.0001, by unpaired Student’s *t* test (**D** and **H**). See also Supplemental Figures 5 and 6.

Beyond their cytoplasmic roles, the MST1/2 kinase domains may shuttle into the nucleus to regulate chromatin condensation (33, 34) and apoptosis (35, 36) after undergoing a caspase-mediated cleavage event to remove their C-terminal domains (37). Nevertheless, full-length MST2 was recently found to also be able to localize either in the inner nuclear membrane (38) or the nucleoli (39). These findings prompted us to speculate that upon DNA damage, MST1/2 kinases may disassociate from STRIPAK complexes and translocate into the nucleus, where they could exert their function of suppressing DNA repair. And indeed, using a cellular compartment fractionation assay, we repeatedly detected increased accumulation of MST1/2 proteins in the nucleus upon etoposide treatment (Figure 5A). Interestingly, the molecular weights of nuclear MST1/2 were the same as those of their cytoplasmic forms (Figure 5A), indicating that full-length kinases, but not the caspase-cleaved products, were translocated into the nucleus.

To determine whether MST1 has a role in the nucleus of restraining DSB repair, and to distinguish such a role from its previously reported functions, we generated a series of truncation and point mutation variants of MST1 (Figure 5B). First, we replaced Asp326/Asp349 with asparagine (referred to herein as 2DN) (40) to create a mutant MST1 resistant to caspase cleavage. Also, we created a truncated form of MST1 (referred to as 1-326) to mimic the residual kinase domain after cleavage. And we replaced the 4 critical leucine residues for the nuclear export signal with alanine (referred to as 4LA [ref. 41]) to generate a mutant MST1 that would be predominantly localized in the nucleus. We then confirmed, using an immunofluorescence assay, that WT and 2DN were mainly localized in the cytoplasm, whereas 1-326 and 4LA were mainly in the nucleus (Figure 5C). Note that the 4LA mutant displayed a better nuclear localization efficiency than did 1-326 (Figure 5C).

Subsequently, we performed DSB reporter assays using MST1-KO cells reconstituted with comparable levels of the indicated MST1 variants (Supplemental Figure 6A). Strikingly, WT and the 2DN mutant each only partially rescued, and the 1-326 and 4LA mutants almost fully rescued, the inhibitory effect of MST1 on the repair capacity of both HR (Figure 5D) and NHEJ (Supplemental Figure 6B). Of note, the suppressive role of 4LA was even stronger than that of 1-326 (Figure 5D), a phenomenon correlating with their nuclear localization efficiencies. And consistent with these observations, the residual γ-H2AX levels 12 hours after IR treatment in 1-326– or 4LA-expressing cells were markedly higher than those in cells expressing WT or the 2DN mutant of MST1 (Figure 5E).

As with MST1, we also generated several MST2 mutants including D322N, 1-322, and 4LA-NLS (Figure 5F). We found that WT MST2 and its D322N mutant were mainly located in the cytoplasm, whereas 1-322 and 4LA-NLS were mainly in the nucleus (Figure 5G). In line with their relative abundances in the nucleus upon DNA damage, the DSB reporter assay (Figure 5H and Supplemental Figure 6, C and D) and our analysis of residual γ-H2AX levels (Figure 5I) revealed that reconstitutions of MST2-KO cells with, respectively, 1-322 or 4LA-NLS more efficiently rescued the inhibitory effect on DNA repair than did the reconstitutions with, respectively, WT and D322N. Taken together, these results indicated that after DNA damage–triggered release from STRIPAK, full-length MST1/2 kinases translocated into the nucleus to suppress DSB repair independently of the classical Hippo signaling and caspase-mediated cleavage event.

### The MST1/2 kinases directly phosphorylate ZMYND8 at Ser486 and Ser490.

**Figure 6:**
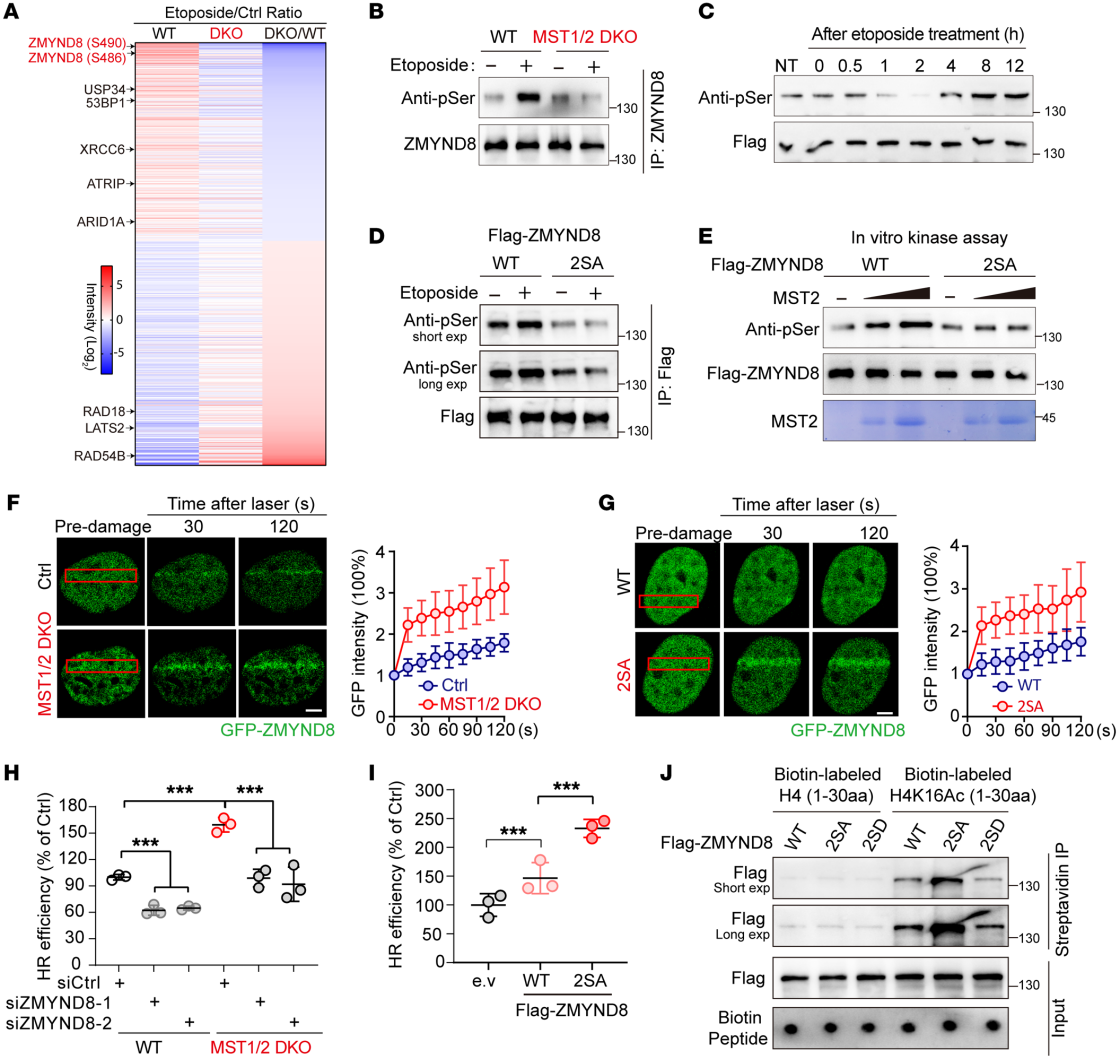
MST1/2 kinases phosphorylate ZMYND8 and limit ZMYND8-dependent DSB repair. (**A**) Heatmap demonstration of the relative abundances of phosphopeptides identified using MS analysis. (**B**) Gels showing attenuation of ZMYND8 phosphorylation in MST1/2-DKO cells. HGC-27 cells (WT and MST1/2-DKO) were treated with etoposide and then harvested 8 hours after treatment. (**C**) Time-resolved analysis of ZMYND8 phosphorylation upon DNA damage in HGC-27 cells. (**D**) Gels showing ZMYND8 phosphorylated at Ser486/Ser490. 293FT cells transfected with the indicated constructs were exposed to etoposide and harvested 8 hours later. (**E**) Gels showing that MST2 phosphorylated ZMYND8 in vitro. Purified Flag-ZMYND8 proteins (WT and 2SA) were incubated with MST2 recombinant protein at 30°C for 15 minutes. (**F** and **G**) Images showing that phosphorylation of ZMYND8 by MST1/2 suppressed its recruitment to DSBs. (**F**) HGC-27 cells (WT and MST1/2-DKO) were transfected with GFP-ZMYND8, or (**G**) HGC-27 cells were transfected with GFP-tagged ZMYND8 or its 2SA mutant before being subjected to laser-induced live cell imaging (*n =* 5). Scale bars: 1 μm. (**H**) Plot showing that depletion of ZMYND8 rescued hyperactive HR repair in MST1/2-deficient cells (*n =* 3). (**I**) Results of the HR reporter assay of 293A cells transfected with WT ZMYND8 or its 2SA mutant (*n =* 3). (**J**) Gels showing that phosphorylation of ZMYND8 by MST1/2 impaired its recognition of H4K16Ac. ***P < 0.001, by 1-way ANOVA with Dunnett’s post hoc test (**H** and **I**). See also Supplemental Figure 7. exp, exposure.

Considering the dependence of the MST1/2 suppression function on their kinase activities, we applied advanced label-free quantitative phosphoproteomics to identify potential substrate(s) through which MST1/2 limit DSB repair in the nucleus. To this end, we treated WT and MST1/2-DKO HGC-27 cells with or without etoposide for 18 hours and then harvested the cells for follow-up MS analysis (Supplemental Figure 7A). Analysis of phosphopeptides that were significantly enriched in WT but not MST1/2-DKO cells revealed the occurrence of several MST1/2-dependent phosphorylation events resulting from the DNA damage (Figure 6A). For example, we noticed that 53BP1 phosphorylation was reduced in MST1/2-deficient cells (Figure 6A), consistent with the results of a laser strip assay (Supplemental Figure 7B), suggesting the validity of our phosphoproteomics results. Intriguingly, at the top of the list were 2 amino acid residues (Ser486 and Ser490) derived from ZMYND8, a critical chromatin remodeling regulator involved in DSB repair (42, 43), which was thus chosen for further analysis.

To validate the MS results, we performed a ZMYND8 IP assay in WT and MST1/2-DKO cells and used an antibody specific for phosphorylated serine (p-Ser) to examine the serine phosphorylation status upon DNA damage. We reproducibly detected a marked etoposide treatment–induced increase in ZMYND8 serine phosphorylation in WT cells, but hardly any such increase in MST1/2-DKO cells (Figure 6B). Interestingly, such serine phosphorylation was reduced in the early stages but gradually increased in the late stages after DNA damage (Figure 6C), a pattern resembling the time-dependent alteration of p-MST1/2 (Figure 2D). Moreover, the MST2-ZMYND8 interaction was also enhanced upon treatment with etoposide (Supplemental Figure 7C). Next, we adopted a point mutation strategy to confirm the occurrence of phosphorylation of ZMYND8 mainly at the 2 identified serine residues. To this end, we replaced each serine with either alanine (referred to herein as 2SA) to generate a ZMYND8 variant defective in MST2-mediated phosphorylation, or with aspartate (referred to herein as 2SD) to mimic constitutive phosphorylation of ZMYND8. Consistent with results with the MST1/2-DKO cells, we observed a DNA damage–triggered increase of ZMYND8 serine phosphorylation in cells expressing WT but not its 2SA mutant for both 293A (Figure 6D) and HGC-27 cells (Supplemental Figure 7D). A further in vitro kinase assay using recombinant MST2 protein and purified Flag-ZMYND8 (WT and 2SA) proteins showed that MST2 could, in a dose-dependent manner, phosphorylate WT ZMYND8 but not its 2SA mutant (Figure 6E), indicating ZMYND8 to be a direct substrate of MST1/2 in the nucleus upon the occurrence of DNA damage.

### Phosphorylation of ZMYND8 by MST1/2 attenuates its recruitment to DSBs.

Given that ZMYND8 promotes HR repair by remodeling the damaged chromatin (42), we reasoned that MST1/2 may suppress DNA repair by impeding the recruitment of ZMYND8 to DSBs. To validate this hypothesis, we first compared the laser microirradiation–triggered accumulations of GFP-tagged ZMYND8 at DSBs between WT and MST1/2-deficient cells. In line with the above hypothesis, we observed a much more robust accumulation of GFP-tagged ZMYND8 in MST1/2-DKO cells than in control WT cells (Figure 6F). Moreover, pretreatment of WT cells with XMU-MP-1, a reported MST1/2 kinase inhibitor (44), similarly resulted in enhanced GFP-ZMYND8 accumulation at DSBs in HGC-27 cells (Supplemental Figure 7E). Meanwhile, the 2SA mutant displayed a much stronger signal of localization at DNA damage tracks than did the WT ZMYND8 (Figure 6G). Functionally, depletion of ZMYND8 using 2 different siRNAs efficiently reversed the hyperactive HR repair capacity in MST1/2-DKO cells (Figure 6H and Supplemental Figure 7F). On the contrary, ectopic expression of the 2SA mutant promoted HR repair more profoundly than did the WT ZMYND8 (Figure 6I).

To further dissect the mechanism through which MST1/2-mediated ZMYND8 phosphorylation limits DSB repair, we performed a chromatin fractionation assay using samples of cells expressing, respectively, WT, the 2SA mutant, and the 2SD mutant of ZMYND8, and found that the 2SA protein level was much higher and the 2SD protein level was lower than the WT ZMYND8 level in the chromatin fraction upon IR treatment (Supplemental Figure 7G). Given that recruitment of ZMYND8 to DSBs requires its recognition of histone H4 acetylated at K16 (H4K16Ac) (42, 43), we synthesized biotin-labeled peptides corresponding to the N-terminus of H4 (denoted as 1–30 aa) with or without the K16Ac modification. Our in vitro pulldown assay revealed that the 2SA mutant, but not the 2SD mutant, had markedly higher binding affinity for the H4K16Ac peptide than did WT ZMYND8 (Figure 6J), suggesting that phosphorylation of ZMYND8 by MST1/2 attenuates its interaction with H4K16Ac, thereby limiting ZMYND8-dependent DSB repair.

### Loss of Hippo signal endows cancer cells with radio-chemoresistance.

**Figure 7:**
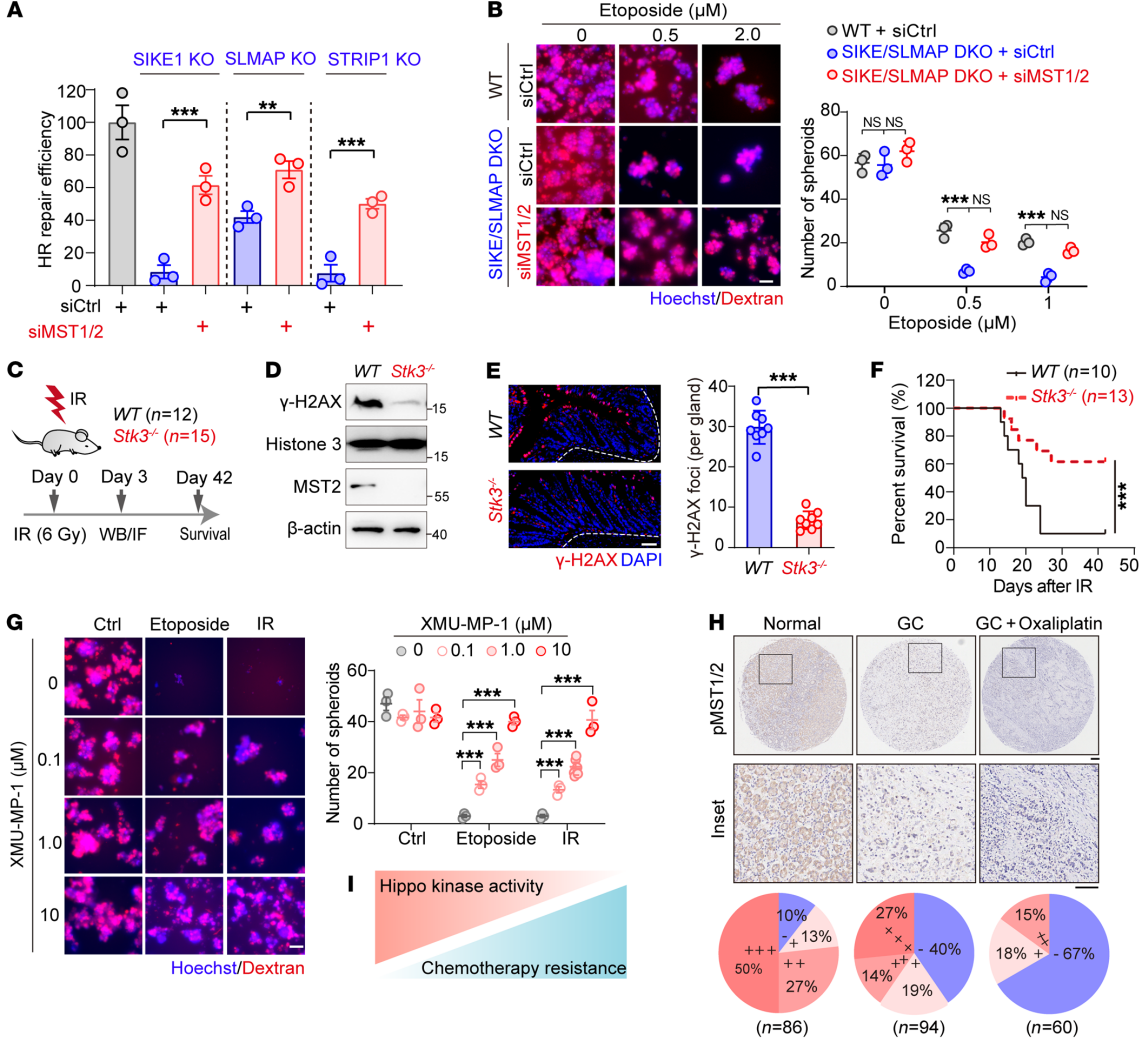
Loss of Hippo kinase activity endows cancer cells with resistance to radio- and chemotherapy. (**A**) Plot showing that MST1/2 ablation restored HR repair in STRIPAK component–deficient cells. 293A cells (WT or KOs) were transfected with the indicated siRNAs before being subjected to an HR reporter assay (*n =* 3). (**B**) Images of samples from HGC-27 cells (WT and SIKE1/SLMAP-DKO) transfected with the indicated siRNAs and then subjected to a sphere formation assay (*n =* 3). Scale bar: 60 μm. (**C**–**F**) Results showing that MST2 deficiency induced radioresistance in vivo. (**C**) WT and *Stk3^–/–^* mice were exposed to 6 Gy whole-body IR. Residual DNA damage in intestinal tissues was examined at 3 days via both (**D**) Western blotting and (**E**) immunofluorescence analysis using anti–γ-H2AX antibody, and (**F**) overall survival was assessed until 6 weeks after IR. Representative images in intestinal tissues were quantified and analyzed (*n* = 8 glands from 2 mice in each group). Scale bar: 10 μm. (**G**) Images showing that MST1/2 inactivation promoted cancer cell resistance to etoposide. HGC-27 cells were treated with etoposide with or without XMU–MP-1 and analyzed using the sphere formation assay (*n =* 3). Scale bar: 60 μm. (**H**) Reduced MST1/2 kinase activity in tumor tissues after chemotherapy. IHC analysis of p-MST1/2 staining of tissues derived from adjacent normal tissue, GC tumors, and GC tumors after oxaliplatin treatment. Scale bar:100 μm, the enlarged inserts were magnified 5 times form original images. (**I**) Schematic illustration of the negative correlation between Hippo kinase activity and acquired drug resistance. **P < 0.01 and ***P < 0.001, by unpaired Student’s *t* test (**A** and **E**) and 1-way ANOVA with Dunnett’s post hoc test (**B** and **G**). The survival analysis was performed using the log-rank test (**F**). See also Supplemental Figure 8.

Considering the hyperactive DNA repair capacity in MST1/2-deficient cells, we speculated that loss of the Hippo signal, which has been frequently observed in cancers (28), may endow cancer cells with resistance to radio- and chemotherapy. Given the established role of STRIPAK in restraining MST1/2 activation, we first validated this hypothesis in the context of the Hippo-containing STRIPAK complex. To this end, we depleted MST1/2 kinases using siRNAs in SIKE-, SLMAP-, and STRIP1-deficient cells and reexamined their DSB repair capacities. As expected, codepletion of MST1/2 kinases efficiently restored otherwise impaired HR repair in these STRIPAK component–deficient cells (Figure 7A and Supplemental Figure 8A). Moreover, in SIKE1-SLMAP–DKO HGC-27 cells, which were hypersensitive to etoposide, further depletion of both kinases strongly restored the sphere-formating ability of these cells to a level comparable to that of WT cells (Figure 7B and Supplemental Figure 8B).

Next, we continued to examine whether deficiency of MST1/2 could enhance DNA repair and radioresistance in vivo. In this regard, it has been shown that global deficiency of both MST1/2 causes embryonic lethality, and that mice lacking MST1 harbor an immunodeficiency syndrome, whereas mice lacking MST2 grow normally with no gross phenotype (37). Since we did not have MST1/2–conditional KO mice at hand, we used MST2-KO mice (*Stk3^–/–^*) for the in vivo radiosensitivity analysis (Figure 7C). Consistently, *Stk3^–/–^* mice showed much lower levels of residual DNA damage (γ-H2AX levels), as revealed by either Western blotting (Figure 7D) or immunofluorescence staining (Figure 7E), in intestinal tissues on day 3 after 6 Gy whole-body IR, indicating a much faster DNA repair in these mice. Accordingly, the *Stk3^–/–^* mice showed more profound radioresistance, i.e., they had a much higher survival rate than did WT mice (Figure 7F).

As an alternative to the depletion strategy, we also tested whether pharmacological targeting of the Hippo kinase activity would affect tumor cell resistance to radio- and chemotherapy. To this end, we treated HGC-27 cells, a GC cell line with relatively high Hippo kinase activity, with various doses of the compound XMU-MP-1 and examined the corresponding sphere-forming abilities of the cells with or without etoposide or IR treatment. Consistent with the notion that loss of Hippo signal stimulates DNA repair and confers chemotherapy resistance, we found that both the numbers (Figure 7G) and diameters of spheroids (Supplemental Figure 8C) gradually increased along with the decrease of MST1/2 kinase activity upon treatment with XMU-MP-1 (Supplemental Figure 8D).

Finally, we examined the clinical relevance of the Hippo signal, i.e., the kinase activity of MST1/2 in chemotherapy resistance. To this end, we performed IHC analysis of p-MST1/2 in tissue microarrays of 86 adjacent normal tissues, 94 GC tumors, and 60 tumor tissues from patients receiving oxaliplatin chemotherapy. We observed significant decreases in p-MST1/2 levels in 23% of the normal tissues (20 of 86), 60% of the GC tissues (56 of 94), and nearly 80% of the oxaliplatin-treated GC tissues (40 of 60), respectively (Figure 7H). In line with the negative regulation of MST1/2 on ZMYND8, this analysis also revealed that the expression levels of ZMYND8 were higher in oxaliplatin-treated tissues than in untreated tumor tissues (Supplemental Figure 8E).

Taken together, these results clearly established a negative correlation between loss of Hippo signal and the acquired radio- and chemotherapy resistance (Figure 7I).

### Rational restoration of MST1/2 kinase activity resensitizes GC cells to PARPi.

**Figure 8:**
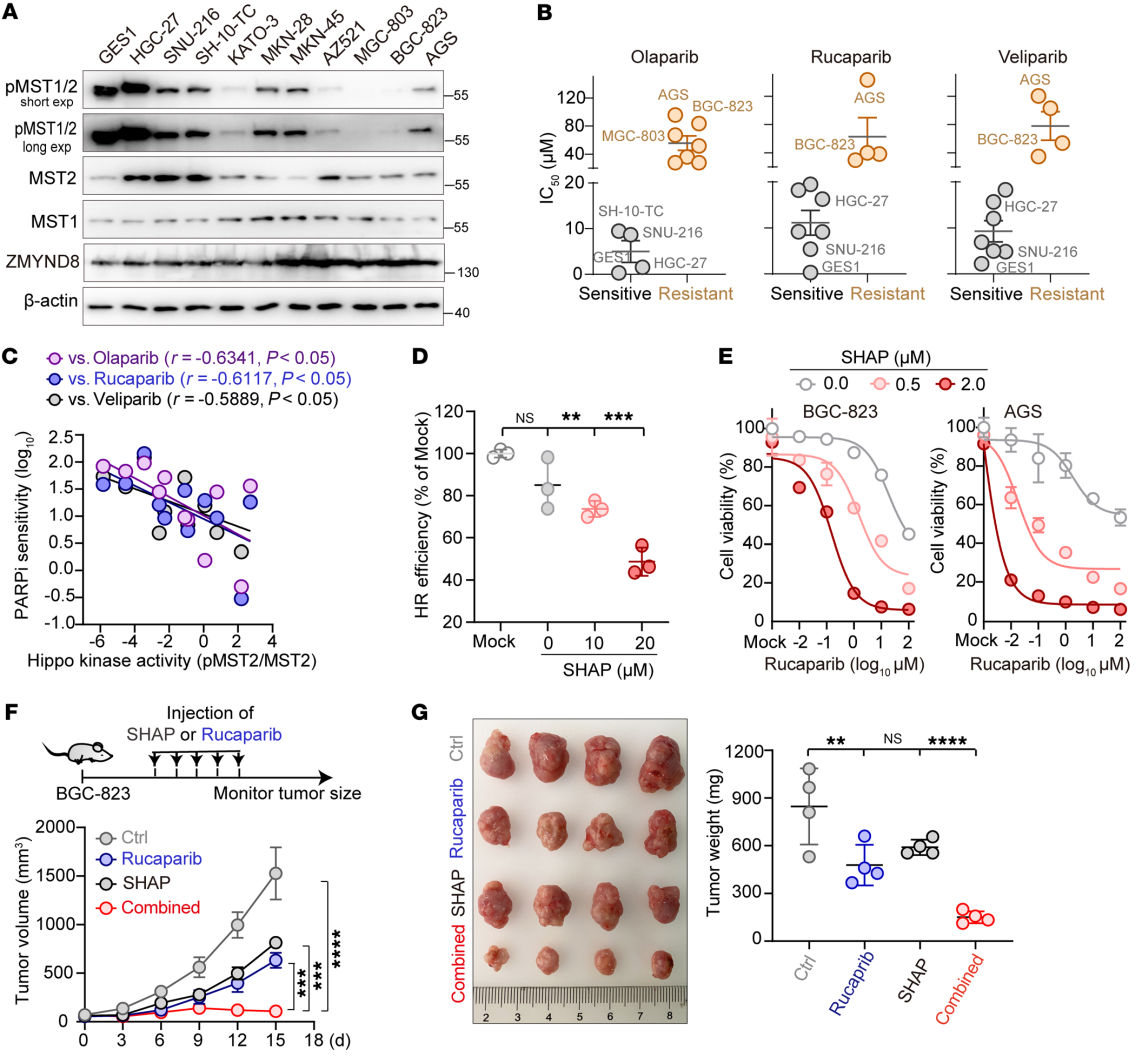
Rational restoration of Hippo kinase activity resensitizes tumors to PARP inhibition. (**A**) Western blot analysis of p-MST1/2 and ZMYND8 across 11 GC cell lines. (**B**) IC_50_ values measured from the GC cell lines after they were treated with each PARPi. A group was defined to be sensitive or resistant to PARPi based on whether the IC_50_ was, respectively, less than or greater than 20 μM. (**C**) Linear regression analysis of PARPi sensitivity and Hippo kinase activity across the GC cells. (**D**) Plot showing a dose-dependent suppression of HR repair by SHAP (*n =* 3). (**E**) Plots showing that treatment of GC cells with SHAP resensitized the cells to PARPi. Specifically, BGC-823 and AGS cells were each treated with various doses of rucaparib in the presence of 0, 0.5, and 2.0 μM SHAP for 24 hours. (**F** and **G**) Results showing that SHAP treatment dramatically enhanced rucaparib-mediated antitumor efficiency in vivo. Nude mice bearing GC tumors were intraperitoneally administered SHAP and rucaparib, either alone or in combination. (**F**) Tumor size was measured every 3 days, and (**G**) tumors were photographed and weighed on day 18 (*n* = 4 mice/group). **P < 0.01, ***P < 0.001, and ****P < 0.0001, by 1-way ANOVA with Dunnett’s post hoc test (**D** and **G** ) and unpaired Student’s *t* test (**F**). See also Supplemental Figure 9.

Since an activated MST1/2/ZMYND8 axis could robustly inhibit DSB repair and loss of this axis induced the development of drug resistance, we next examined whether such is also the case in GC for PARPi. To this end, we first examined the Hippo kinase activity (p-MST1/2) and ZMYND8 expression levels in cells from 11 GC cell lines. We found that p-MST1/2 levels were much higher in some cell lines, such as HGC-27 and SNU-216, than in others, such as AGS and BGC-823 (Figure 8A). Next, we measured the IC_50_ values of 3 FDA-approved PARPi drugs, namely olaparib, rucaparib, and veliparib, in these GC cells. On the basis of the IC_50_ values, we divided the cell lines into PARPi-sensitive (IC_50_ <20 μM) or PARPi-resistant (IC_50_ >20 μM) groups (Figure 8B). Interestingly, we found that the cells with relatively high p-MST1/2 levels, such as HGC-27 and SNU-216 cells, were relatively sensitive to PARPi, whereas those harboring relatively low levels of p-MST1/2, including MGC803 and AGS cells, were relatively resistant to PARPi (Figure 8B). Linear regression analysis revealed a significant negative correlation between Hippo kinase activity and PARPi resistance (Figure 8C), whereas ZMYND8 expression was positively correlated with PARPi resistance (Supplemental Figure 9A).

To validate the correlation between the Hippo kinase activity and PARPi resistance, we used XMU-MP-1 to inactivate MST1/2 kinases in HGC-27 cells and found that these cells became much more resistant to PARPi (Supplemental Figure 9B). Confirming the results obtained in HGC-27 cells, we found that codepletion of SIKE1 and SLMAP in SNU-216 cells dramatically attenuated their sphere-forming ability upon treatment of the cells with etoposide (Supplemental Figure 9C), whereas the sphere-forming ability was increased in a dose-dependent manner following the inactivation of MST1/2 kinase activity by XMU-MP-1 (Supplemental Figure 9D). Similarly, SNU-216 cells pretreated with XMU-MP-1 also acquired resistance to PARPi (Supplemental Figure 9E).

As the Hippo signal is frequently lost in GC, we reasoned that an artificial increase of MST1/2 kinase activity with the SHAP peptide acting as an agonist would resensitize these tumors to PARP inhibition. Consistent with the aforementioned inhibitory effect of MST1/2 on DNA repair, we found that SHAP treatment attenuated the HR repair capacity in a dose-dependent manner (Figure 8D). Importantly, although BGC-823 and AGS cells were resistant to PARPi because of the extremely low Hippo activity in these cells, cotreatment with SHAP was found, remarkably, to resensitize both types of cells to both rucaparib (Figure 8E) and olaparib (Supplemental Figure 9F) treatments.

We also investigated the combined tumor-killing potency of PARPi and SHAP in a xenograft mouse model. Briefly, we first injected mice subcutaneously with BGC-823 cells for 1 week to incubate tumors, and then delivered intraperitoneally either rucaparib or SHAP, or a combination of both, for 4 consecutive days and monitored tumor size (Figure 8F). While rucaparib treatment yielded modestly lower tumor weights and growth rates than did the control treatments, cotreatment of rucaparib and SHAP almost completely blocked tumor growth (Figure 8, F and G), suggesting that restoration or elevation of MST1/2 kinase activity can resensitize tumors to PARPi.

### Cotargeting of STRIPAK and PARP elicits synthetic lethality in tumor cells.

**Figure 9:**
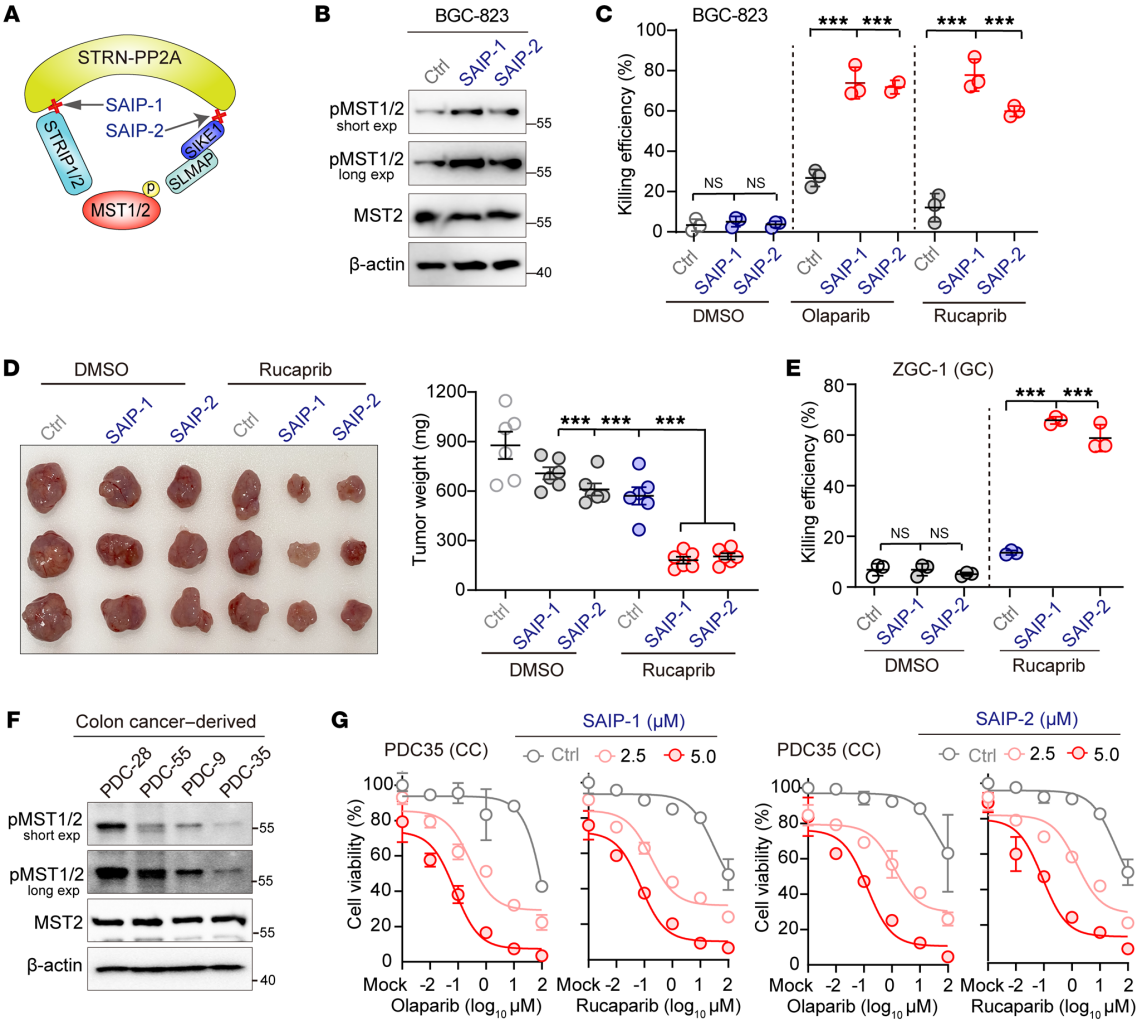
Cotargeting of STRIPAK and PARP elicits synthetic lethality in tumor cells. (**A**) Schematic illustration of the STRIPAK assembly peptide inhibitors SAIP-1 and SAIP-2. (**B**) Gels showing that inclusion of SAIP-1 and SAIP-2 each restored Hippo kinase activity in BGC-823 cells. (**C**) Plots showing that SAIP1/2 in combination with PARPi resulted in synthetic lethality in BGC-823 cancer cells (*n =* 3). (**D**) Photograph showing that SAIP-1/2 synergistically augmented rucaparib-mediated antitumor efficiency in vivo (*n* = 3 mice/group in 1 experiment; *n =* 2 assays). Dot plot represents mouse tumor weights from 2 experiments (*n* = 6 mice per group) in each group. (**E**) Plots showing that SAIP-1/2 had a synthetic lethality effect with PARPi in gastric PDCs with low Hippo activity (*n =* 3). (**F** and **G**) Gels and plots showing that SAIP-1/2 had a synthetic lethality effect with PARPi in colon PDCs with low Hippo activity. (**F**) Western blot analysis of p-MST1/2 levels in 4 colon cancer PDC lines. (**G**) PDC35 cells were further treated with various doses of olaparib or rucaparib in the presence of 0, 2.5, and 5.0 μM SAIP-1 (left panel) or SAIP-2 (right panel) for 24 hours before cell viability analysis. CC, colon cancer. ***P < 0.001, by 1-way ANOVA with Dunnett’s post hoc test (**C**, **D**, and **E**). See also Supplemental Figure 10.

Inspired by the SHAP-induced resensitization of cancer cells to PARPi, we further explored the possibility of targeting the STRIPAK assembly to induce defects in DNA repair, specifically by activating the MST1/2 kinases, which would induce synthetic lethality when combined with PARPi. To this end, we chose to target the interaction of STRN3 with SIKE1 and also with STRIP1, as these 2 “molecular arms” are crucial for the recruitment of MST1/2 into STRIPAK (7) and exert profound stimulating effects on DNA repair (Figure 1). Guided by the crystal structure of the STRN3-SIKE1 subcomplex (PDB: 6AKL) and the detailed biochemical characterization of the STRN3-STRIP1 interaction (7), we synthesized 2 peptides based on the STRN3 interaction interface with either STRIP1 or SIKE1 and named them STRIPAK assembly inhibitor peptide 1 and 2 (SAIP-1 and SAIP-2), respectively (Figure 9A and Supplemental Figure 10A). In vitro pulldown assays clearly demonstrated that SAIP-1 could directly bind to STRN3 (Supplemental Figure 10B) and that SAIP-2 disrupted the STRN3-SIKE1 interaction in a dose-dependent manner (Supplemental Figure 10C). Moreover, a co-IP assay using BGC-823 cells showed the abilities of SAIP-1 and SAIP-2 to efficiently disrupt the interactions of STRN3 with STRIP1 and SIKE1-SLMAP, respectively (Supplemental Figure 10D). Importantly, treatment here with SAIP-1 or SAIP-2 robustly increased p-MST1/2 levels (Figure 9B), indicating the suitability of our targeting strategy.

To assess whether SAIP-1/2 can induce synthetic lethality in GC when combined with PARPi, we determined the IC_50_ values of SAIP-1/2, olaparib, and rucaparib individually in BGC-823 cells and used nontoxic concentrations for further combination analysis. Strikingly, although treatments with SAIP-1 or SAIP-2 alone and PARPi alone each exhibited a moderate efficiency at killing cancer cells, combining SAIP-1 and SAIP-2 with either olaparib or rucaparib induced the deaths of nearly 70% of tumor cells, showing a strong effect of synthetic lethality (Figure 9C). Moreover, a further in vivo xenograft mouse model recapitulated such synergistic tumor-suppressive effects when combining rucaparib with either SAIP-1 or SAIP-2 (Figure 9D and Supplemental Figure 10E).

Finally, we moved on to further evaluate our proposed synthetic lethality therapy in patient-derived cells (PDCs). We first chose gastric PDCs of the cell line ZGC-1, which have a diminished Hippo signal (12), and determined for ZGC-1 the IC_50_ values of olaparib, rucaparib, and SAIP-1/2 (Supplemental Figure 10F) as described above. Consistent with the above results, a combined nontoxic dose of SAIP-1/2 with rucaparib or olaparib strongly induced synthetic lethality in ZGC1 cells, resulting in the death of more than 60% of the cells (Figure 9E and Supplemental Figure 10G). In addition, we also isolated and generated 4 colon PDC lines (PDC-9, PDC-28, PDC-35, and PDC-55) and simultaneously examined their sensitivity to PARPi and the phosphorylation levels of MST1/2 in these cells. In line with observations in GC cells, we found that PDC-9 and PDC-35 harbored relatively lower Hippo kinase activity (Figure 9F), and displayed resistance to PARPi (Supplemental Figure 10H). Importantly, treatment with SAIP-1 or SAIP-2 reproducibly resensitized both PDC-9 and PDC-35 to PARPi in a dose-dependent manner (Figure 9G and Supplemental Figure 10I).

Taken together, these results demonstrated that targeting of STRIPAK could recover the kinase activity of MST1/2, which, in combination with PARPi, could elicit strong synthetic lethality in human gastrointestinal tumors.

## Discussion

In the past decade, emerging evidence has indicated that STRIPAK acts as a stress sensor that orchestrates extracellular stimuli to initiate intracellular processes (6, 7, 9, 18). In addition to extracellular stress, here we showed that STRIPAK could also respond to genotoxic stress as a result of its dynamic assembly and active tuning of the MST1/2 kinases, which ultimately regulate nuclear DNA repair and tumor cell sensitivity to anticancer therapies. Mechanistically, the cGAS/STING pathway relays the DNA damage signal to STRIPAK immediately after exposure of a cell to genotoxic stress, using a machinery in which activated TBK1 binds to and stabilizes the SIKE1-SLMAP arm to achieve a tighter STRIPAK-MST1/2 complex. As such, the MST1/2 kinases are better recruited into and inactivated by the STRIPAK complex, which relieves the inhibitory effect of MST1/2 on ZMYND8 recruitment to DSBs, thereby allowing for efficient DSB repair and endowing cancer cells with resistance to radio- and chemotherapy (Supplemental Figure 11A). In this regard, the STRIP1 component of STRIPAK has recently been reported to regulate chemotherapy sensitivity via p21 (45). Taken together, these pieces of evidence have established an essential role for STRIPAK in regulating the repair of DNA damage and genomic stability, adding a new layer to the STRIPAK-associated functional network.

Although it might seem inefficient for cells to deploy cytoplasmic signaling cascades to urgently cope with chromatin DNA damage stress, intensive studies have uncovered a complex crosstalk between cytoplasmic signaling and responses to nuclear DNA damage (46, 47). Here, we found that, upon sensing DNA damage, cGAS/STING/TBK1 signaling could result in stabilization of the SIKE1-SLMAP arm and thus tighten the STRIPAK complex to eventually inactivate the MST1/2 kinases and allow for the recruitment of ZMYND8 to DSBs. Considering the quick mobilization of ZMYND8 to laser strips and its functionality as a chromatin remodeling factor (42), all these events actually take place as early as 1 hour after the occurrence of DNA damage. Thus, using the on-site cGAS-STING DNA sensor machinery and a cytoplasm-to-nucleus regulatory circuit such as STRIPAK-MST1/2-ZMYND8 is actually an efficient way for cells to cope with such urgent genotoxic stress. That said, as the orchestration of genomic instability and innate immunity by the cGAS/STING pathway canonically occurs several days after radio- or chemotherapy (20), it currently remains elusive how this signaling cascade can induce the STRIPAK assembly within such a short amount of time after DNA damage. In this regard, noncanonical roles of the cGAS/STING pathway are emerging (48). For example, cytosolic cGAS can be recruited to DSB loci within several minutes (49), and etoposide treatment can trigger innate immune responses within 4 hours via a cGAS-independent STING/TBK1 signaling cascade (50). Overall, these pieces of evidence suggested that the cGAS/STING signaling may also play important roles in the early sensing of DNA damage.

The MST1/2 kinases have been well established to play vital roles in balancing cell proliferation (via YAP/TEAD4; refs. 28, 29) and apoptosis (via YAP/p73; refs. 23–27). Beyond their traditional cytoplasmic tumor-suppressive function, our current study revealed a nonclassical YAP-independent role for the MST1/2 kinases: they can enter in a form without caspase-mediated cleavage into the nucleus to suppress DNA repair via direct phosphorylation of the chromatin remodeling factor ZMYND8. Intriguingly, during the preparation of our manuscript, the Lou group reported that the MAP4K4/5/6 kinases, which are the functional paralogs of MST1/2 in the Hippo pathway and STRIPAK complexes, can limit DSB repair capacity and mediate cancer cell sensitivity to genotoxic agents at low extracellular stiffness conditions (51). Importantly, the authors similarly revealed a YAP-independent regulatory mechanism for MAP4K4/5/6 in DSB repair control. Overall, it appears that components of the Hippo pathway can modulate the DSB repair capacity and tune cancer cell drug resistance not only in a classical YAP-dependent manner but also in a nonclassical YAP-independent manner.

Distinct from the previously reported nuclear functions of MST1/2 that are mediated by their kinase domains upon apoptosis-induced caspase cleavage (34–37, 40, 41), our current work has shown that full-length MST1/2 kinases can shuttle into the nucleus to directly suppress DSB repair. In this regard, caspase-mediated MST1/2 cleavage and the subsequent nuclear translocation of their kinase domain usually occur in the late stages (>24 h) upon persistent apoptotic stimulation (34–37, 40, 41). In contrast, we only treated cells with DNA-damaging reagents for 1 to 2 hours to induce genome-wide DNA damage and further tracked the repair events after removal of the reagents, a scenario mainly concerning DSB repair in the early stages of DNA damage, yet without substantial apoptosis. Of note, the Eric O’Neill group recently reported that a nuclear fraction of full-length MST2 can directly bind chromatin to regulate transcriptional repression at early time points after DNA damage (39). Based on all of these observations, we proposed a model involving dual regulatory roles of MST1/2 kinases in response to DNA damage: full-length MST1/2 kinases are freed from STRIPAK and translocate into the nucleus to phosphorylate ZMYND8 and therefore restrain its recruitment to DSB loci, suppressing DSB repair in the early stages; but MST1/2 kinases can also undergo caspase-mediated cleavage resulting in nuclear translocation of their kinase domains to initiate apoptosis in the late stages.

A number of upstream regulators of the Hippo pathway, including STRIPAK (18, 52), RASSF1A (24, 26, 53), and FAT1 (54), have been found to be dysregulated. For example, hypermethylation of the *RASSF1A* promoter region has been found in almost all human solid tumors (55). Such dysregulations usually result in reduced or lost Hippo tumor suppressor activity. In this regard, we recently developed SHAP to recover the lost Hippo signal for cancer treatment (12). In addition to restraining cancer cell proliferation, here we also showed that SHAP-induced recovery of MST1/2 kinase activity could suppress DSB repair and resensitize GC cells to PARP inhibition. Furthermore, we developed 2 additional STRIPAK-targeting peptide inhibitors, namely SAIP-1 and SAIP-2, and showed that they could also efficiently restore the kinase activity of MST1/2, suppress DNA repair, and elicit synthetic lethality when combined with PARPi (Supplemental Figure 11A). Together with the use of SHAP, we have achieved 3 different approaches to recovering Hippo kinase activity via targeting of STRIPAK, opening new opportunities for a combination therapy with current antitumor strategies.

Notably, PARPi have not been approved for treating gastrointestinal cancers. In a previously conducted mutational signature analysis, only 7% to 12% of patients with GC harbored intrinsic DNA repair defects (56), which may partially explain the poor efficacy of olaparib for the treatment of patients with advanced GC in clinical trials (57). To improve the clinical benefits of PARPi, we found that targeting STRIPAK to recover or even enhance MST1/2 kinase activity could induce the development of DNA repair defects, regardless of the intrinsic DNA repair machinery, and therefore resensitize cancer cells to PARP inhibition (Supplemental Figure 11B). Importantly, we expect such induced synthetic lethality via simultaneous targeting of STRIPAK and PARP to be applicable to all loss-of-Hippo tumors, regardless of cancer type.

In summary, our work reveals that cytoplasmic STRIPAK, via the cGAS/STING/TBK1 pathway, serves as a sensor for nuclear genotoxic stress and further reveals a STRIPAK/MST1/2/ZMYND8 axis involved in regulating DNA repair and genomic stability, shedding new light on the sophisticated crosstalk between cytoplasmic signaling and the nuclear DNA damage response. Moreover, we established that reduced Hippo kinase activity endows cancer cells with resistance to radio- and chemotherapy and demonstrated that cotargeting of STRIPAK and PARP to induce synthetic lethality can serve as an anticancer strategy.

## Methods

A detailed description of the materials and methods used are provided in the Supplemental Methods.

### Study approval.

The approval ID for the use of animals was SIBCB-NAF-14-004-S329-023, issued by the Animal Core Facility of the Shanghai Institute of Biochemistry and Cell Biology. All clinical samples used in this study were derived from patients under the approval of the ethics committee of Taizhou Hospital (Zhejiang Province, China). Detailed clinical information was collected from all patients.

## Author contributions

Z Zhou, SJ, and LA designed the research. LA and ZC performed most cellular experiments with input from Z Zhao, QZ, and YY. PN, HZ, FC, and ZT conducted the in vivo assays. YT and GF designed and synthesized the peptide inhibitors. LA and ZC analyzed the data with input from YH and WW. YX, LS, and YY provided key reagents. Z Zhou, SJ, LA, and ZC wrote the manuscript. Z Zhou, SJ, HM, and HX supervised the project.

## Supplementary Material

Supplemental data

## Version 1 03/15/2022

In-Press Preview

## Version 2 05/02/2022

Electronic publication
