# Supplementary material for: Combinatorial targeting of Hippo-STRIPAK and PARP elicits synthetic lethality in gastrointestinal cancers
Source: J Clin Invest. 2022 May 2;132(9):e155468. doi: 10.1172/JCI155468 (PMC9057599; doi:10.1172/JCI155468)
Supplement: Supplemental data [file jci-132-155468-s209.pdf]

Supplementary Information

Combinatorial targeting of Hippo-STRIPAK and PARP elicits synthetic lethality in gastrointestinal cancers

Liwei An, Zhifa Cao, Pingping Nie, Hui Zhang, Zhenzhu Tong, Fan Chen, Yang Tang, Yi Han, Wenjia Wang, Zhangting Zhao, Qingya Zhao, Yuqin Yang, Yuanzhi Xu, Gemin Fang, Lei Shi, Huixiong Xu, Haiqing Ma, Shi Jiao, Zhaocai Zhou

Supplementary Materials and Methods

Supplementary Figures 1-9

Figure S1. Hippo-containing STRIPAK complex is essential for DSB repair and genomic stability

Figure S2. The MST1/2 kinases suppress DSB repair in a manner dependent on their kinase activity

Figure S3. The STRIPAK-MST1/2 undergoes dynamic assembly in response to DNA damage

Figure S4. The cGAS-STING-TBK1 pathway relays DNA damage signal to STRIPAK assembly

Figure S5. MST1/2 kinases suppress DSB repair independent of LATS-YAP signaling

Figure S6. MST1/2-mediated suppression of DSB repair requires their nuclear localization

Figure S7. MST1/2 kinases phosphorylate ZMYND8 to limit DSB repair

Figure S8. Reduced Hippo kinase activity confers cancer cell drug resistance

Figure S9. Resistance of cancer cells to PARPi is associated with reduced Hippo kinase activity

Figure S10. Co-targeting STRIPAK and PARP elicit synthetic lethality in human tumors

Figure S11. A working model for MST1/2-mediated restriction of DSB repair and antitumor strategy

Supplementary Tables

Table S1. List of siRNA sequence used in this manuscript

Materials and methods

Cell culture

Cells of human HEK293FT, 293A and gastric cancer cell lines including AGS, AZ-521, BGC-823, MGC-803, and HGC-27 cells were obtained from the cell library of the Chinese Academy of Sciences (Shanghai, China). SNU-216, KATO-III, MKN-45, SH-10-TC and GES1 cells were purchased from ATCC and the RIKEN BioResource Centre, respectively. All of the cancer cell lines including the PDCs were maintained in RPMI 1640 medium (Invitrogen). HEK293FT, 293A, and the knockout cells derived from these cell lines were cultured in DMEM medium (Invitrogen). Cells were grown in culture medium supplemented with 10% FBS, 100 μg/mL penicillin, and 100 μg/mL streptomycin in 5% CO_2 at 37°C with saturated humidity. Chemicals used in this work, including KU-55933 (ATM inhibitor, S1092), VE-821 (ATR inhibitor, S8007), XMU-MP-1 (MST1/2 inhibitor, S8334), RU.521 (cGAS inhibitor, S6841), C-176 (STING inhibitor, S6575), MRT67307 (TBK1 inhibitor, S7948), AA-673 (TBK1 inhibitor, S3648), olaparib (S1060), rucaparib (S4948), and veliparib (S1004), were all purchased from Selleck.

Plasmids

For constructs transiently expressed in mammalian cells including the Flag-tagged MST1, MST2, and ZMYND8 constructs as well as their related mutants, were sub-cloned into a modified pCDNA-3.1 vector with an N-terminal 3xFlag tag. Specifically, SIKE1-expressing plasmids including WT-, M1 mutant (E96A/R109A/A100D/M105E), M2 mutant (L15D/L19D/L36D), and 6SE mutant (S185E/S187E/S188E/S190E/S198E/S203E) were inserted into an N-terminal HA-tagged pCDNA-3.1 vector. SLMAP-expressing plasmids including WT-,△FHA mutant (deletion of residues 5–153), and 4LD mutant (L182D/L186D/L189D/L193D) were cloned and expressed in a pCDH1-MCS-coGFP vector. For the laser micro-irradiation assay, sequences of WT- and 2SA mutant (S486A/S490A) of ZMYND8 were transferred into a pEGFP-C1 vector with an N-terminal GFP tag. For constructs for BioID assay, the genes of interest were constructed into a PCDH-EF1a-BirA vector. For bacterium-mediated protein expression and purification of MST2 (1–320aa) and STRN3 (64–341aa), constructs were cloned into a modified pET-28a vector with an N-terminal MBP tag. Full-length SIKE1 and STRN3 (64–135aa) were engineered into a pET-28a

vector with an uncleavable C-terminal His6 tag. All mutants were generated by performing site-directed mutagenesis and were verified by performing Sanger sequencing.

Transfection, lentivirus packaging, and infection

For experiments involving RNAi-mediated depletion, cells were transfected with two rounds of either nontargeting control or targeting siRNAs (GenePharma) using Lipofectamine RNAiMAX Transfection Reagent (13778150, Invitrogen) according to the manufacturer's instructions. The sequences of siRNAs used in this study are listed in **Supplementary Table 1**. For experiments involving plasmid-based transfection, constructs were first incubated with polyethylenimine (PEI, 23966-1, Polysciences) at a ratio of 1:3 for 15 minutes at room temperature, and then the resulting mixture was administered dropwise into the above-described culture medium. Lentiviral particles were produced by co-transfection of the lentiviral-based constructs together with packaging of plasmids psPAX2 and pMD2.G at a mass ratio of 4:3:1 into HEK293FT cells. At 48 h after transfection, the supernatants were filtered (0.45 μm) and were applied to recipient cells in the presence of 8 μg/ml polybrene (H9268, Sigma Aldrich).

CRISPR-Cas9 approach for the generation of KO cells

All of the KO cell lines used in this study were generated using the CRISPR-Cas9 gene-targeting approach. Briefly, sequences of two guide RNAs (gRNAs) targeting genes of interest were obtained from the human GeCKO library (https://www.genscript.com/CRISPR-gRNA-library.html), and were sub-cloned into the pLentiCRISPRv2 vector (52961, Addgene). The sequences of gRNAs used in this study were as follows: SIKE1 gRNA#: 5'- CACCGAGCATCCTGGTGTTCCTCCA-3'; SLMAP gRNA#: 5'-CACCGATACTCACTCTGAGCGGAGC-3' STRIP1 gRNA#: 5'-CACCGAAAGATGTTGTAGCGCATCC-3'; STRIP2 gRNA#: 5'- CTACCTCATACCTGACAACT-3'; MST1 gRNA#: 5'-AGCTTTGTATACGCTGCCAT-3'; and MST2 gRNA#: 5'-ACAGCAACGAGAATTGGAAG-3'. For the generation of KOs, cells were infected with lentiviral particles harboring indicated gRNAs twice at 24-h intervals. KO cells were selected by applying 1 μg/ml puromycin for one week before validation was performed by carrying out western blotting using indicated antibodies.

Immunoprecipitation and western blotting

For whole-cell extracts, cells were lysed with 20 mM Tris·HCl, pH 8.0, 100 mM NaCl, 0.5% NP-40, and 1 mM EDTA (NETN buffer) supplemented with micrococcal nuclease (1:5000, M0247S, NEB) on ice for 15 minutes. Cell lysates were boiled with 5× SDS loading buffer, separated using SDS-PAGE, transferred to polyvinylidene fluoride membranes, and immunoblotted with indicated antibodies. The antibodies used and their dilutions were as follows: MST1 (#3682, CST, 1:1000), MST2 (ab52641, Abcam, 1:1000), Phospho-MST1 (Thr183) / MST2 (Thr180) (referred as pMST1/2, #3681, CST, 1:500), SIKE1 (ab121860, Abcam, 1:1000), SLMAP (sc-393336, Santa Cruz, 1:500), STRIP1 (ab199851, Abcam, 1:1000), STRIP2 (HPA019657, Atlas Antibodies, 1:200), PP2A A subunit (#2041, CST, 1:1000), PP2A Catalytic alpha subunit (#2038, CST, 1:1000), YAP (#14074, CST, 1:1000), TAZ (ab224239, Abcam, 1:1000), anti-HA (H3663, Sigma, 1:2000), anti-Flag (F3165, Sigma, 1:2000), β-actin (#A5441, Sigma, 1:5000), ZMYND8 (11633-1-AP, Proteintech, 1:1000), γ-H2AX (ab229914, Abcam, 1:2000), histone H3 (ab1791, Abcam, 1:5000), and phospho-Ser antibody (sc-81514, Santa Cruz, 1:1000). The secondary HRP antibodies including goat anti-rabbit (31460, 1:4000), and goat anti-mouse (31430, 1:4000) were purchased from Thermo Fisher Scientific. Western blot images were captured by using the Mini Chemiluminescent Imaging and Analysis System (Beijing Sage Creation Science Co, LTD).

For Co-IP experiments, cells were lysed with NETN buffer for 15 minutes on ice, with the resulting material subjected to centrifugation at 12,000 rpm for 10 minutes at 4 °C. Supernatants were then transferred into new Eppendorf tubes and incubated with either Flag beads (L00425, GenScript) for 4 h or Protein A/G Plus Agarose beads (sc-2003, Santa Cruz) conjugated with indicated antibodies overnight at 4 °C with stirring. The resulting beads were subsequently washed three times with NETN buffer and boiled with 1 x SDS loading buffer. To avoid the noise of light/heavy chains, secondary antibodies were used for the immunoprecipitation assays, with these secondary antibodies including mouse anti-rabbit IgG LCS (A25022, 1:5000) and goat anti-mouse IgG LCS (A25012, 1:5000), which were obtained from Abbkine.

RNA extraction, reverse transcription, and qPCR

Total RNA from cultured cells were extracted using the RNA isolator Total RNA Extraction Reagent (R401-01-AA, Vazyme), and cDNA was reverse-transcribed from

RNA using the HiScript II Q Select RT SuperMix for qPCR (R223-01, Vazyme) according to the manufacturer's instructions. Quantitative real-time PCR was performed using SYBR Green reagents in a Step OnePlus system (Applied Biosystems). Primers used in this study were as follows:

SIKE1 forward: 5'-GGCTTACATCCCTCATGCTGT-3';
SIKE1 reverse: 5'-ATAGCTGGCACAGGCCATTT-3';
SLMAP forward: 5'-TCAGGAGCGTCATGTCTACCT-3';
SLMAP reverse: 5'-CGTGGTTCCTTGATAGCACTTTG-3';
LATS1 forward: 5'-CACTGGCTTCAGATGGACACAC-3';
LATS1 reverse: 5'-GGCTTCAGTCTGTCTCCACATC-3';
LATS2 forward: 5'-GTTCTTCATGGAGCAGCACGTG-3';
LATS2 reverse: 5'-CTGGTAGAGGATCTTCCGCATC-3';
CTGF forward: 5'-AAAAGTGCATCCGTACTCCCA-3',
CTGF reverse: 5'-CCGTCGGTACATACTCCACAG-3';
CYR61 forward: 5'-GGTCAAAGTTACCGGGCAGT-3',
CYR61 reverse: 5'-GGAGGCATCGAATCCCAGC-3';
PUMA forward: 5'-TGAAGAGCAAATGAGCCAAACG-3'
PUMA-reverse: 5'-CAGAGCACAGGATTCACAGTCT-3'
Bax forward: 5-CCTGTGCACCAAGGTGCCGGAACT-3
Bax reverse: 5-CCACCCTGGTCTTGGATCCAGCCC-3
Bcl-2 forward: 5-TTGTGGCCTTCTTTGAGTTCGGTG-3
Bcl-2 reverse: 5-GGTGCCGGTTCAGGTACTCAGTCA-3
GAPDH forward: 5'-GGCATCCTGGGCTACACTGA-3', and
GAPDH reverse: 5'-GAGTGGGTGTCGCTGTTGAA-3'.

DSB reporter assay

293A cells were electroporated with the I-SceI expression construct (pCBASce) together with GR-GFP or EJ5-GFP reporter plasmid at 150 V, 975 microfarads using an NEPA21 Super Electroporator (NEPA GENE, Japan). Cells were further recovered for 48 h after electroporation and were subjected to flow cytometric analysis using a BD FACSCantoII analyzer.

Cell viability assay

For cell death and viability assays, cells were seeded into 96-well plates at a density of 1000 per well overnight to have them become attached to the wells, and then treated with siRNAs, PARPi or PDPA-delivered peptides at indicated concentrations for 48 h. The PDPA micelles were incubated with peptides at 4℃ for at least 1 h. The final concentrations of PDPA and peptides were 500 mg/ml and 100 mg/ml, respectively. The cells were incubated for the indicated length of time, and then used with an ATP-based CellTiter-LumiTM Plus kit (Beyotime) according to the manufacturer's instructions. The intracellular ATP contents were measured using a BioTek Synergy TM NEO multi-detector microplate reader (Thermo). Cell viability was calculated using the equation % Cell viability = [[value (test) - value (blank)] × [value (control)-value (blank)]] -1×100.

Neutral comet assay

Briefly, for each experiment, cells were subjected to 10 Gy of IR and collected at either 0.5 h or 4.0 h post IR. Cells were harvested and rinsed twice with ice-cold PBS, and about 1 x 10^5 cells/ml were mixed with molten LMAgarose (4250-050-02, Enzo) at a ratio of 1:10 (v/v) and immediately a volume of 75 µL of the mixture was pipetted onto a Comet Slide apparatus (4250-050-03, Enzo). Each slide with the mixture was put into a 4°C refrigerator for 30 minutes and then treated with neutral lysis buffer (2.5 M NaCl, 100 mM Na_2EDTA, 1% sodium lauroyl sarcosine, 10 mM Tris, pH 7.5, 1% Triton-X100 and 10% DMSO) overnight. Next, each treated slide was subjected to electrophoresis at 25 V for 30 minutes and stained in CYGREEN® Nucleic Acid Dye agent (ENZ-GEN105-0020, Enzo) for 30 minutes. Images were captured using a fluorescence microscope (Olympus BX51) and analyzed with CometScore software.

3D sphere-formation assay

Single-cell suspensions were suspended in a Dulbecco's modified Eagle's medium/F12 (DMEM/F12, Hyclone, USA) supplemented with 20 ng/mL epidermal growth factor (EGF, Peprotech, USA), and 20 ng/mL basic fibroblast growth factor (bFGF, Peprotech, USA), and then plated in 24-well ultra-low-attachment plates (Corning, USA) at a concentration of 1000 cells per well. Cells were treated with DMSO or various concentrations of indicated chemical drug in the following day. Plates were analyzed 5–7 days later for tumor sphere formation and the spheres were quantified using an

inverted microscope (Olympus). The viable cells were stained with Hoechst 33342 (6249, Thermo Fisher Scientific) and dextran (D1951, Thermo Fisher Scientific).

Laser micro-irradiation and live-cell imaging

HGC-27 cells were grown overnight on a 35-mm glass-bottom dish (D35-20-1-N, Cellvis) and then transfected with ZMYND8 WT or 2SA mutant construct, which were maintained with 10 mM BrdU (B9285, Sigma) for 24 h. Cells were stained with Hoechst 33342 (6249, Thermo Fisher Scientific) for 15 minutes and washed three times with new medium. Next, the cells were placed into a cell culture chamber (37℃, 5% CO2) on an inverted microscope (Eclipse Ti, Nikon). Micro-irradiation was carried out by scanning the regions of interest 20 times using 20% of the 405-nm-wavelength laser power controlled using NIS-Elements software. Images were acquired every 30 s for 10 minutes and exported as TIFF files.

***In vitro* kinase assays**

ZMYND8 proteins (WT- and 2SA mutant) were first purified from HEK293FT cells transiently transfected with indicated constructs for 48 h using Flag beads. Proteins were further incubated with purified recombinant MST2 proteins (1–300aa) in buffers containing ATP for 30 minutes at 30°C with rotation. Samples were boiled with 2 x SDS loading buffer and analyzed by performing western blotting using indicated antibodies.

Colony formation assay

Briefly, cells were seeded and treated with etoposide or IR as indicated in 6-well plates (600 cells per well), and then cultured for 1-2 weeks until visible colonies formed. The colonies were then washed with PBS, fixed, and stained with 0.1% crystal violet for 20 minutes. The plates were then washed with distilled water and air-dried. Visible colonies were counted, and surviving fractions were calculated by comparing the number of colonies formed from the irradiated cells with the number of colonies formed from non-irradiated (control) cells.

BioID proteomics mass spectrum

293A cells expressing indicated constructs were pre-treated with etoposide to induce DNA damage, and then incubated with medium supplemented with 200 μM biotin (B4639, Sigma). The cells were then harvested at indicated times and lysed in NETN

buffer. Resulting supernatants were subjected to immunoprecipitation using streptavidin beads (SA021005, Smart-life Sciences) for 2 h at 4°C. Samples were washed with NETN three times and then processed for mass spectrum analysis (Majorbio, Shanghai).

Pulldown assay using biotin-labeled peptides

Briefly, biotin-labeled peptides corresponding to the N-terminus of H4 (1–30aa) with or without the H4K16ac modification were synthesized by Top-Peptide Biotechnology (Shanghai), and dissolved in a PBS solution. For the pulldown assay, equal concentration of peptides were incubated with cell lysates derived from 293FT cells expressing Flag-tagged ZMYND8 (WT and mutants) for 2 h at 4 °C with rotation. Then, a volume of about 40 μL of streptavidin beads was added to the mixture and then allowed to sit for another 2 h to capture the biotin-conjugated complex. The samples were washed with NETN buffer three times before being boiled with 1xSDS loading buffer. The peptide input was then verified by performing a dot blot.

In vivo mouse model assays

Healthy male BALB/c A-nu/nu mice (3–4 week) were obtained from the Shanghai Experimental Animal Center and maintained in pathogen-free conditions in accordance with the guidelines of the Institutional Animal Care and Use Committee of the Institute of Biochemistry and Cell Biology (SIBCB, Shanghai). The approval ID for the use of animals was No. SIBCB-NAF-14-004-S329-023 issued by the Animal Core Facility of SIBCB.

For mice radio-sensitivity assay, *WT* and $Stk3^{-/-}$ mice were exposed to 6Gy whole-body irradiation and were breed with normal diet. Two mice from each group were sacrificed for immunofluorescence assay and western blotting analysis to examine the residual γ-H2AX levels. The rest of mice were continued to culture for survival analysis and eventually sacrificed at 6 weeks post irradiation.

For the tumor formation assay, mice were first injected with 5×10^6 BGC-823 cells/per mouse into their flanks to induce tumor formation. Once the tumor volume reached about 100 mm^3, mice were injected intratumorally with indicted inhibitors for five successive days. Tumor length and width were measured every 3 days to calculate the tumor volume (= $width^2$ x length x 0.523). Two weeks later, the mice were

euthanized and then their tumors were harvested and weighed.

Immunohistochemistry (IHC) staining

All clinical samples used in this study were derived from patients approved by the Ethics Committee of Taizhou Hospital of Zhejiang province, China. Detailed clinical information was collected from all patients. Tissue microarray sections were prepared by Outdo Biotech (Shanghai, China). For IHC staining, tumor microarray sections were incubated with pMST1/2 antibody (ab79199, 1:50 dilution) and ZMYND8 antibody (ab201452, 1:25 dilution). Their relative expression levels were classified as negative (-), low (+), moderate (++), high (+++), and extremely high (++++) based on visual inspection.

Statistical analysis

The presented data were calculated as mean ± SEM from three independent experiments unless otherwise indicated and were analyzed with GraphPad Prism 8.0 statistical software. Continuous data were compared using Student's *t* tests (comparing two variables) or one-way ANOVA analysis with Dunnett's post hoc test (comparing multiple variables). For correlation, the Spearman rank correlation was used for continuous variables. Survival curves were calculated according to the Kaplan–Meier method; survival analysis was performed using the log-rank test. $p < 0.05$ was considered statistically significant.

Study approval

The approval ID for the use of animals was No. SIBCB-NAF-14-004-S329-023 issued by the Animal Core Facility of SIBCB. All clinical samples used in this study were derived from patients approved by the Ethics Committee of Taizhou Hospital of Zhejiang Province, China.

Supplementary Figures

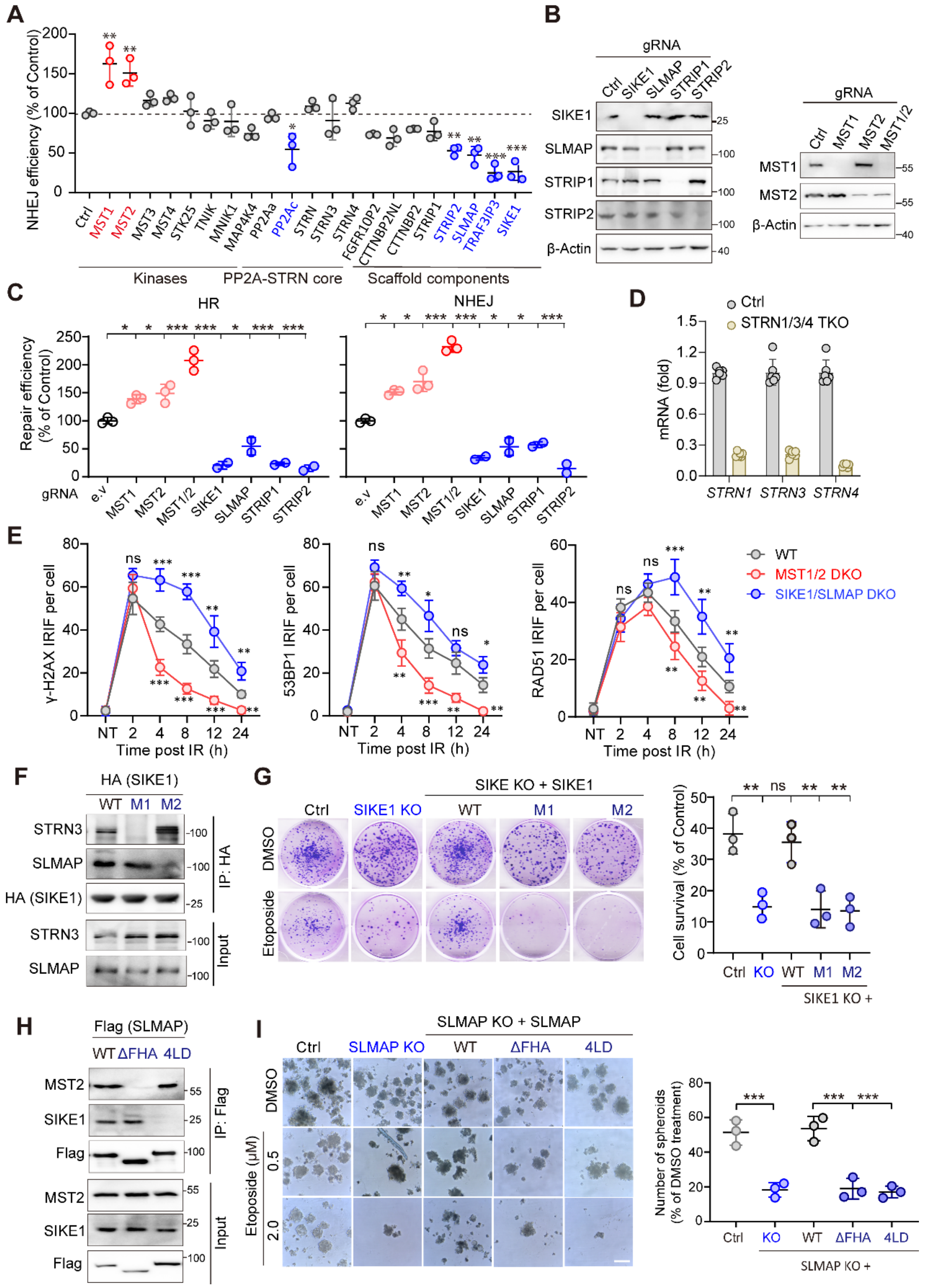

Figure S1. The Hippo-containing STRIPAK complex is essential for DSB repair and genomic stability. (**A**) Plot showing the regulation of NHEJ repair by siRNA-mediated knockdown components of STRIPAK. (**B**) Western blot validation of the knockout cell lines generated using CRISPR-Cas9 technology. (**C**) Results of applying HR and NHEJ reporter assays to 293A cells (WT and indicated KOs). (**D**) qPCR analysis of the STRN1/3/4 triple-knockout cells. (**E**) Plot showing the foci

number γ-H2AX, 53BP1 and RAF51 in each cell line at indicated time points after IR. HGC-27 cells (WT, MST1/2 DKO, and SIKE1/SLMAP DKO) were subjected to 10 Gy IR and fixed at indicated time points before immunofluorescence staining using corresponding antibodies. (**F**) Assay results for co-immunoprecipitation (Co-IP) of HA-tagged SIKE1 (WT and mutants) with endogenous STRN3 or SLMAP in 293FT cells. (**G**) Clonogenic formation assay to evaluate the cell viability of SIKE1 KO cells reconstituted with the indicated constructs after treating the cells with the genotoxic agent etoposide. (**H**) Assay results for Co-IP of Flag-tagged SLMAP (WT and mutants) with endogenous MST2 or SIKE1 in 293FT cells. (**I**) Results of an assay assessing the sphere-formation abilities of SIKE1 KO cells reconstituted with indicated constructs after they were treated with etoposide. For all of the quantification analyses, the presented data were calculated as mean ± SEM from three independent experiments, ns, not significant, *$p < 0.05$, **$p < 0.01$, ***$p < 0.001$; one-way ANOVA with Dunnett's post hoc test, compared with Ctrl.

Related to **Figure 1**

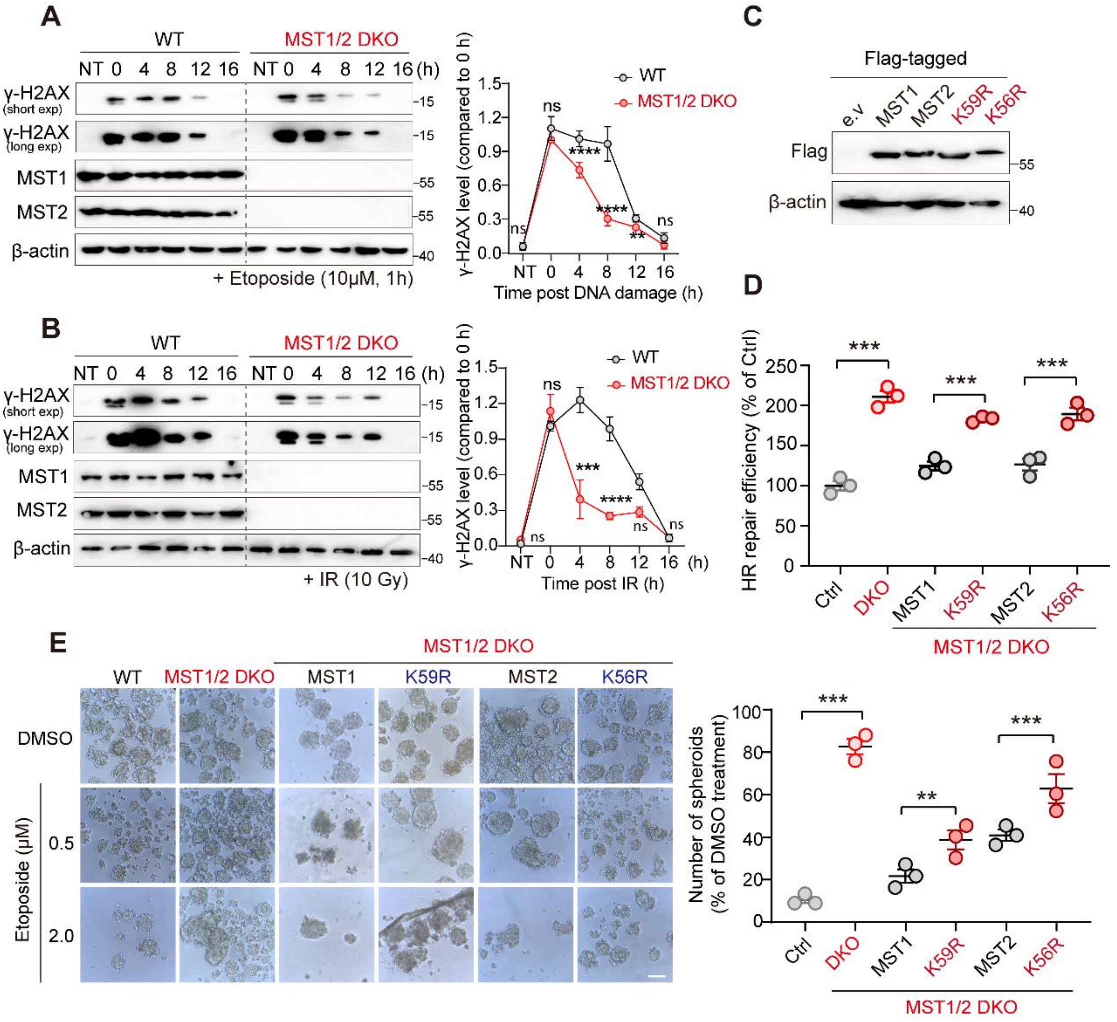

Figure S2. The MST1/2 kinases suppress DSB repair in a manner dependent on their kinase activity. (**A–B**) Western blot analysis of the DSB repair dynamic between WT and MST1/2 DKO HGC-27 cells after induction of DNA damage by either etoposide (A) or IR (B). (**C**) Western blot analysis of MST1/2 DKO cells stably reconstituted with each construct. (**D**) Plot showing the regulation of HR repair in WT and MST1/2 DKO cells expressing indicated constructs using the HR reporter assay (n = 3, ****p* < 0.001; unpaired t test). (**E**) Image and plot of an assay assessing the sphere-formation abilities of MST1/2 DKO cells reconstituted with indicated constructs after they were treated with 0.5 and 2.0 µM etoposide (n = 3, ****p* < 0.001; unpaired t test).

Related to **Figure 1**

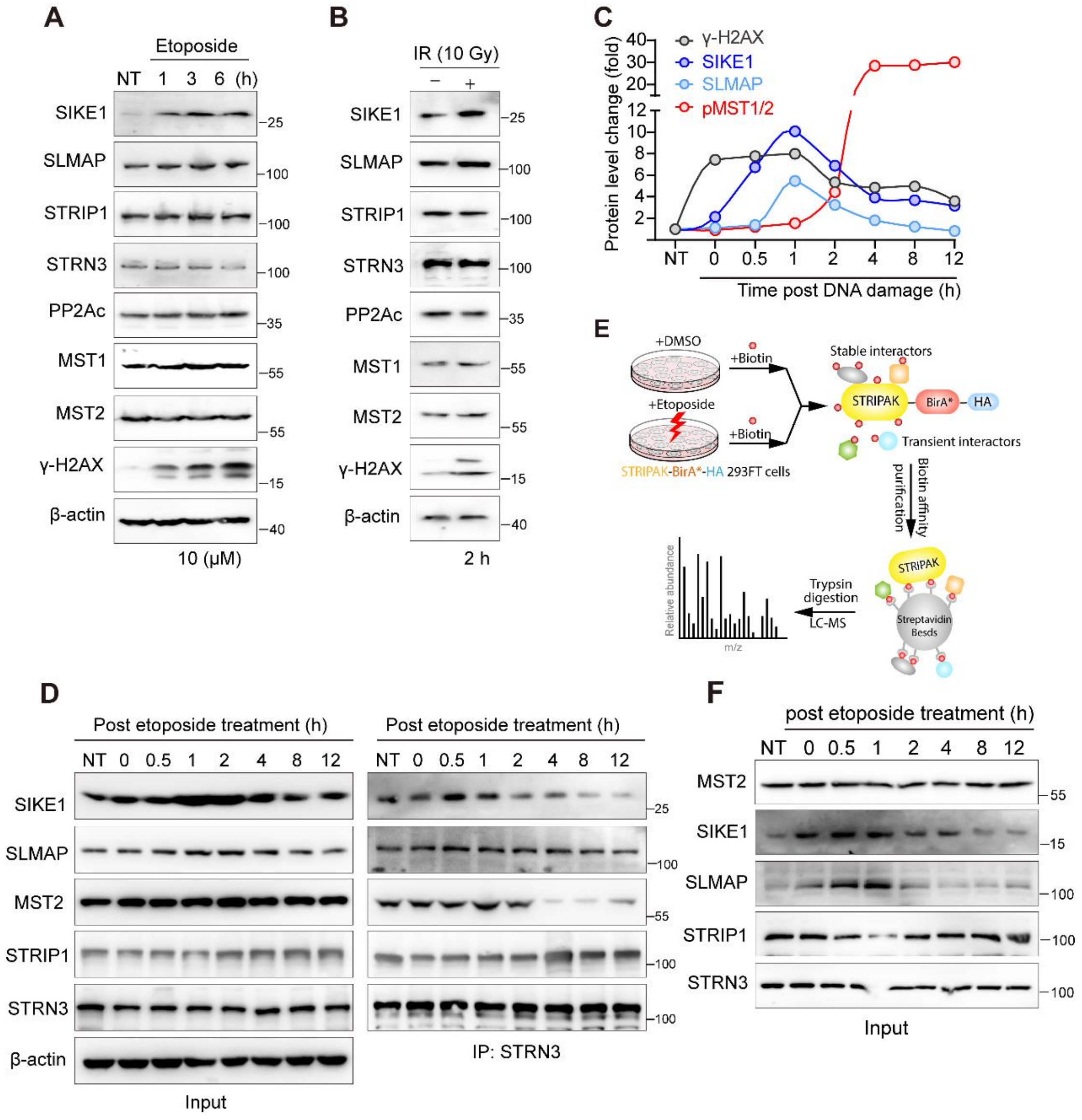

Figure S3. **STRIPAK-MST1/2 undergoes dynamic assembly in response to DNA damage.** (**A–B**) Western blot analysis of the levels of STRIPAK component proteins in response to treatment of 293A cells with either etoposide (A) or IR (B) at 2 h post treatment. (**C**) Plot of quantification of protein levels for SIKE1, SLMAP, pMST1/2 and γ-H2AX at different time points upon induction of DNA damage in 293A cells. **(D)** HGC-27 cells pretreated with 10 μM etoposide for 1 h were collected at indicated time points after removal of the drug for subsequent STRN3 Co-IP analysis. (**E**) Schematic representation of the BioID proteomic assay used to analyze the assembly of STRIPAK-MST1/2 complex upon induction of DNA damage in cells. (**F**) Western blot analysis of the input samples of the Co-IP assay post DNA damage as described in Fig. 2G.

Related to **Figure 2**

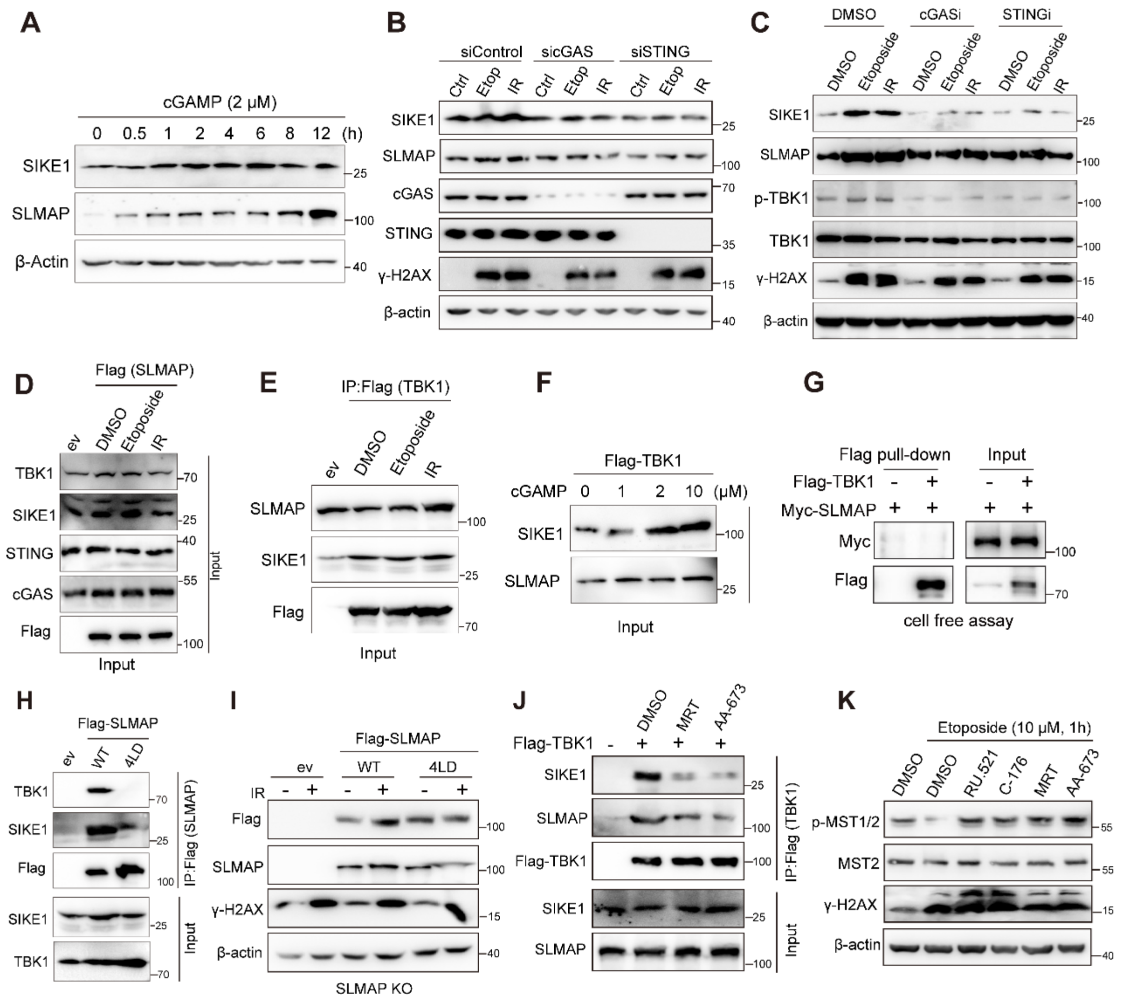

Figure S4. **The cGAS-STING-TBK1 pathway relays DNA damage signaling to STRIPAK assembly.** (**A**) Western blot analysis of SIKE1 and SLMAP each at various time points post cGAMP treatment. (**B–C**) Western blot of indicated proteins after individual depletion of cGAS or STING in HGC-27 cells (B) or chemical inhibition of their kinase activity in 293A cells (C) treated with etoposide or IR. Cells were harvested at 2 h after treatment. (**D–F**) Western blot analysis of the input expression for Co-IP assays in Fig. 4A (D), Fig. 4C (E), and Fig. 4D (F). (**G**) Gels showing a failure of TBK1 to interact with SLMAP in vitro. A Flag pull-down assay was performed using Myc-SLMAP and Flag-TBK1 proteins purified using an in vitro transcription strategy. (**H**) Gels showing interaction of SLMAP with TBK1 via SIKE1. 293FT cells transfected with Flag-tagged WT- or its 4LD mutant were processed for Flag Co-IP assay. (**I**) Plot showing that DNA-damage-induced SLMAP protein stabilization required SLMAP-TBK1 association. 293A SLMAP KO cells reconstituted with either WT or TBK1-binding-defective 4LD mutant were treated with 10 Gy IR and then harvested at 2 h post treatment. (**J**) Gels showing the dependence of the association of TBK1 with SIKE1/SLMAP on its kinase activity. HGC-27 cells transfected with Flag-TBK1 for 30 h were treated with MRT (10 μM) or AA-673 (50 μM) for another 12 h before being examined using the Flag Co-IP assay. (**K**) Gels showing inhibition of the cGAS-STING-TBK1 pathway having failed to inactivate MST1/2 kinase activity at an early stage after induction of DNA damage. HGC-27 cells treated with indicated inhibitors were subjected to 10 Gy IR and harvested at 2 h post IR for subsequent blotting analysis.

Related to **Figure 3 and Figure 4**

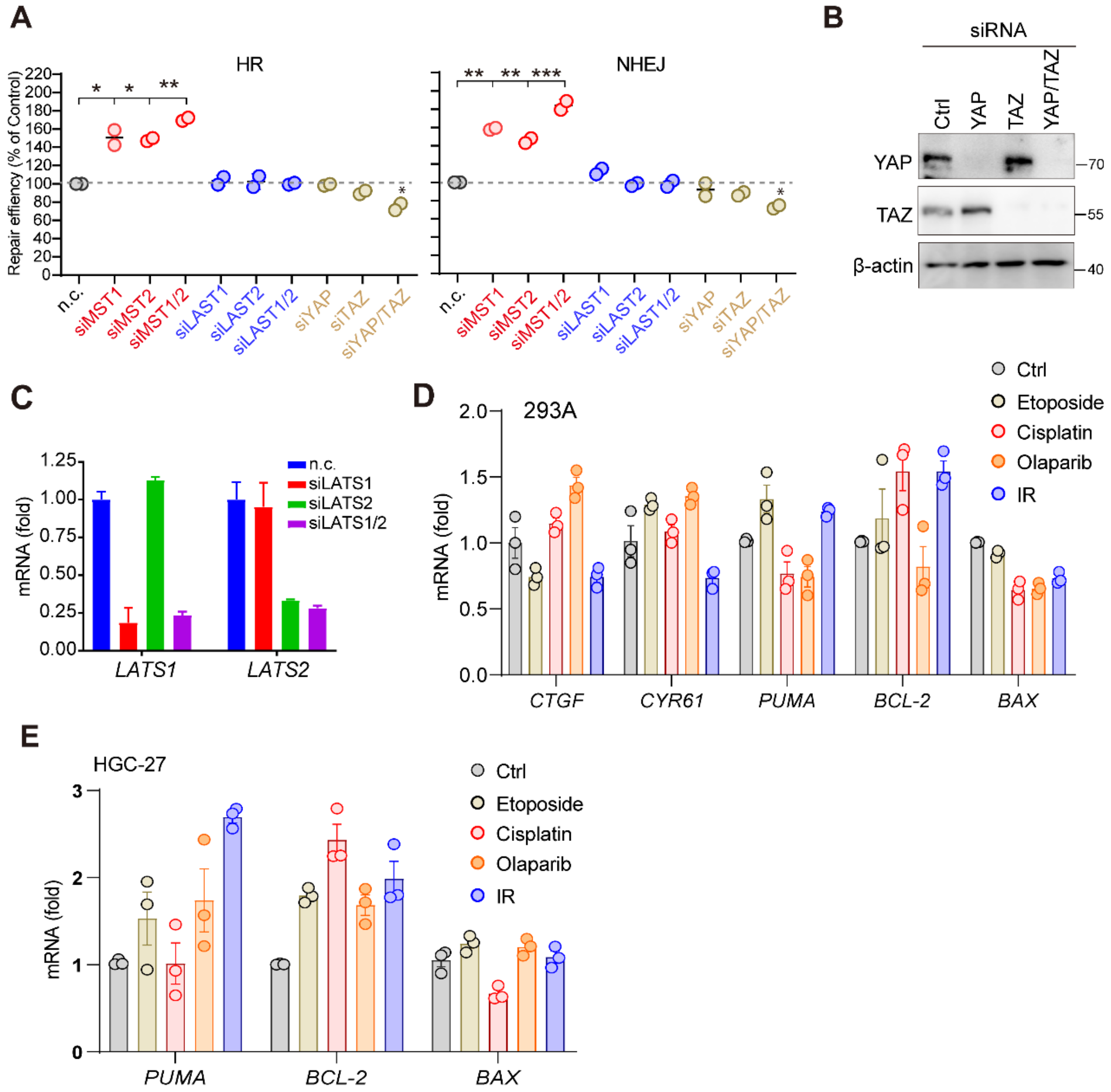

Figure S5. MST1/2 kinases suppress DSB repair independent of LATS1/2-YAP signaling. (**A**) Plot showing the results of subjecting 293A cells transfected with indicated siRNAs to HR and NHEJ reporter assays (n = 3, *p < 0.05, **p < 0.01, ***p < 0.001; one-way ANOVA with Dunnett's post hoc test, compared with Ctrl). (**B**) Western blot analysis of the YAP and TAZ knockdown efficiencies for cells used in A. (**C**) qPCR analysis of the *LAST1* and *LAST2* knockdown efficiencies for cells used in A. (**D–E**) qPCR analysis of the transcription of YAP/TEAD4 target genes *CTGF* and *CYR61*, as well as YAP/p73 target genes including *PUMA*, *BCL-2*, and *BAX*, in response to diverse DNA-damaging agents in both 293A (D) and HGC-27 cells (E).

Related to **Figure 5**

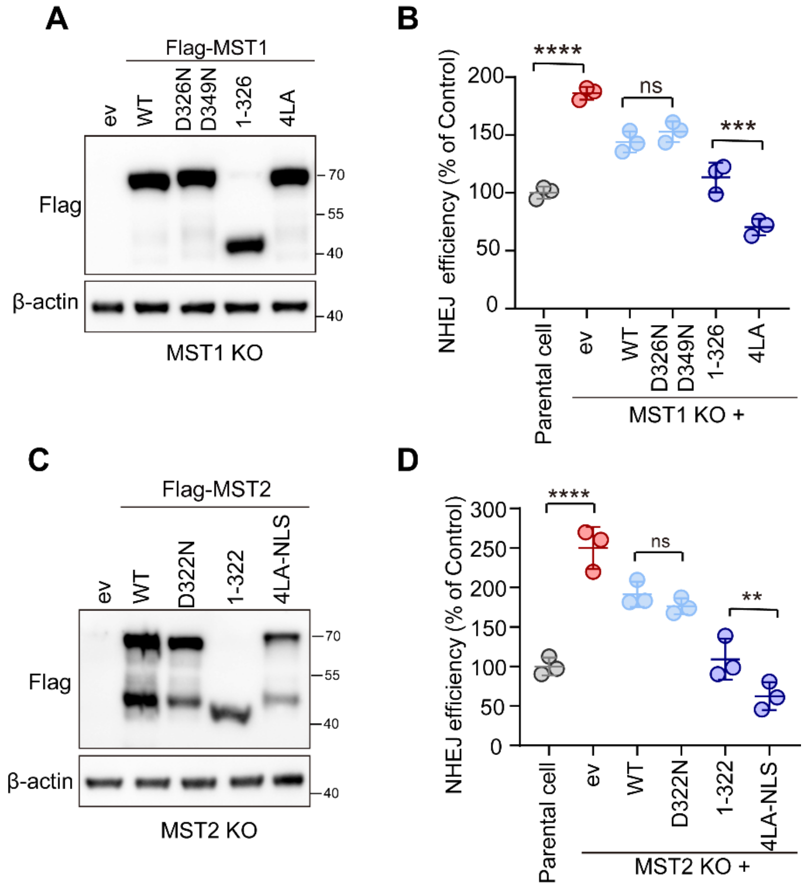

Figure S6. MST1/2-mediated suppression of DSB repair requires nuclear localization of MST1/2. (**A–B**) Gels and plot showing nuclear MST1 having suppressed DSB repair. 293A MST1 KO cells reconstituted with comparable protein level of each MST1 construct (A) were subjected to the NHEJ reporter assay (B) (n = 3, ***$p < 0.001$, ****$p < 0.0001$; unpaired t test). (**C–D**) Gels and plot showing nuclear MST2 having suppressed DSB repair. 293A MST2 KO cells reconstituted with comparable protein level of each MST2 construct (C) were subjected to the NHEJ reporter assay (D) (n = 3, ns, not significant, **$p < 0.01$, ****$p < 0.0001$; unpaired t test).

Related to **Figure 5**

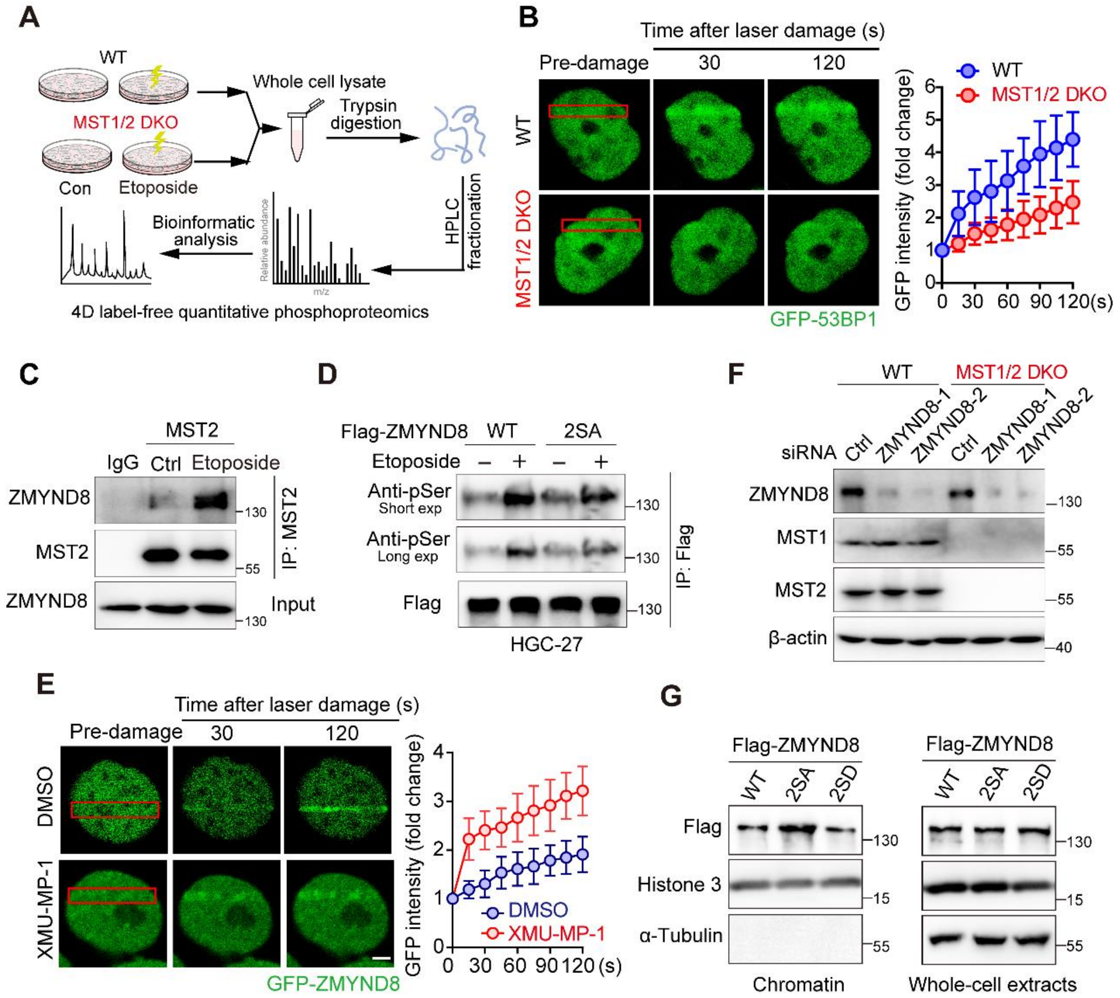

Figure S7. MST1/2 kinases phosphorylate ZMYND8 to limit DSB repair. (**A**) Schematic representation of the 4D label-free phosphoproteomics analysis performed in HGC-27 WT and MST1/2 DKO cells. (**B**) Images showing MST1/2 kinases having promoted 53BP1 recruitment to DSBs. HGC-27 cells (WT and MST1/2 DKO) were transfected with GFP-53BP1 for 30 h before being visualized using laser-induced live cell imaging. Relative GFP fluorescence intensity at laser-induced stripes were quantified (n = 5). (**C**) Gels showing DNA damage having promoted an association of MST2 with ZMYND8. HGC-27 cells were treated with or without etoposide for 12 h before being processed for an MST2 immunoprecipitation assay. (**D**) Gels showing ZMYND8 having been phosphorylated at S486/S490 upon the occurrence of DNA damage. HGC-27 cells transiently transfected with indicated constructs were exposed to etoposide for 12 h before being processed for a Flag immunoprecipitation assay. (**E**) Images showing MST1/2 kinase inhibition having promoted ZMYND8 recruitment to DSBs. HGC-27 cells transfected with GFP-ZMYND8 for 30 h were pretreated with 10 μM XMU-MP-1 for 2 h before processed as in B. (**F**) Western blot showing the siRNA-mediated knock down efficiency of ZMYND8 in HGC-27 cells. (**G**) Gels showing phosphorylation of ZMYND8 by MST1/2 having impaired its association with chromatin. HGC-27 cells were transfected with indicated Flag-tagged ZMYND8 constructs for 36 h and then exposed to 10 Gy IR. Cells were harvested at 8 h post treatment and subjected to a chromatin fractionation assay.

Related to **Figure 6**

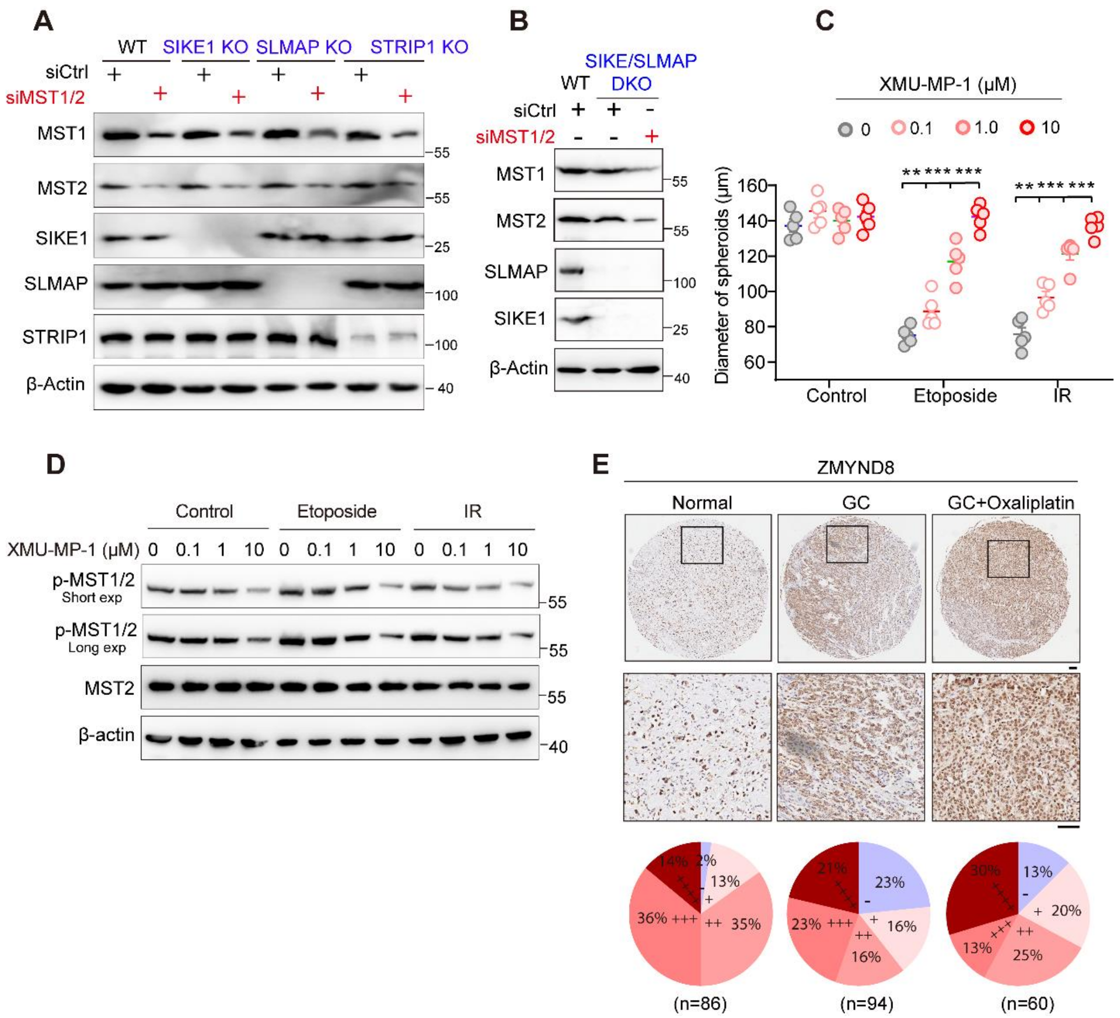

Figure S8. Reduced Hippo kinase activity endows cancer cells with drug resistance. (**A**) Western blot analysis of the knockdown efficiencies of MST1/2 in SIKE1-, SLMAP-, and STRIP1 knockout 293A cells. (**B**) Western blot analysis of the knockdown efficiencies of MST1/2 in SIKE1/SLMAP DKO HGC-27 cells. (**C**) Quantification of spheroid size in HGC-27 cells treated first with the indicated dose of XMU-MP-1 and then with etoposide or IR. (**D**) Western blot analysis of the dose-dependent inhibitory effect of XMU-MP-1 on MST1/2 kinase activity in HGC-27 cells. (**E**) Representative IHC images showing the levels of ZMYND8 on tissue microarrays derived from 86 adjacent normal tissues, 94 GC tumors, and 60 tumor tissues from GC patients who underwent oxaliplatin chemotherapy treatment (Scale bar, 100 μm). Protein level staining of ZMYND8 in each group were quantified. Negative (-), low (+), moderate (++), high (+++), and extremely high (++++) expression levels.

Related to **Figure 7**

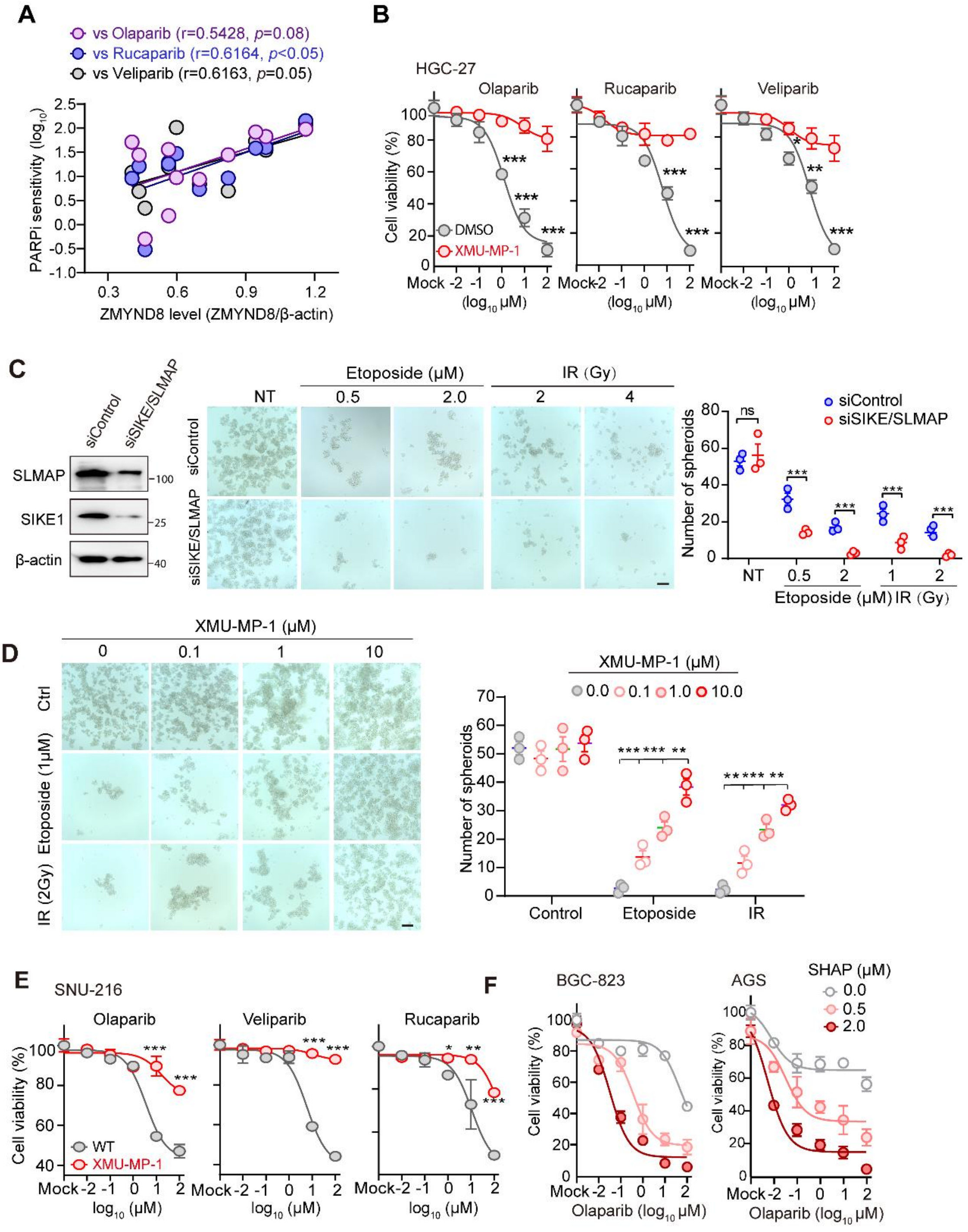

Figure S9. **Resistance of cancer cells to PARPi is associated with reduced Hippo kinase activity.** (**A**) Linear regression analysis revealed positive correlations between ZMYND8 upregulation and PARPi sensitivity ($Log_{10}IC50$). (**B**) Plots showing inactivation of MST1/2 by XMU-MP-1 having induced PARPi resistance in HGC-27 cells (n = 3, ****p* < 0.001; unpaired t test). (**C**) Gels, images and plot showing that co-depletion of SIKE1/SLMAP impaired sphere-formation ability of SNU-216 cells exposed to DNA-damaging IR or etoposide (n = 3, ns, not significant, ****p* < 0.001; unpaired t test). (**D**) Images and plot showing that inactivation of MST1/2 by XMU-MP-1 promoted sphere-formation ability of SNU-216 cells exposed to IR or etoposide (n = 3, ns, ***p* < 0.01, ****p* < 0.001; one-way ANOVA with Dunnett's post hoc test, compared with DMSO). (**E**) Plots showing inactivation of MST1/2 having induced PARPi resistance of SNU-216 cells(n = 3, ****p* < 0.001;

unpaired t test). (**F**) Plots showing SHAP treatment having re-sensitized GC cells to PARPi in vitro. Samples of BGC-823 and AGS cells were each treated with various doses of olaparib in the presence of, respectively, 0, 0.5, and 2.0 μM SHAP for 24 h before being tested for cell viability (n = 3).

Related to **Figure 8**

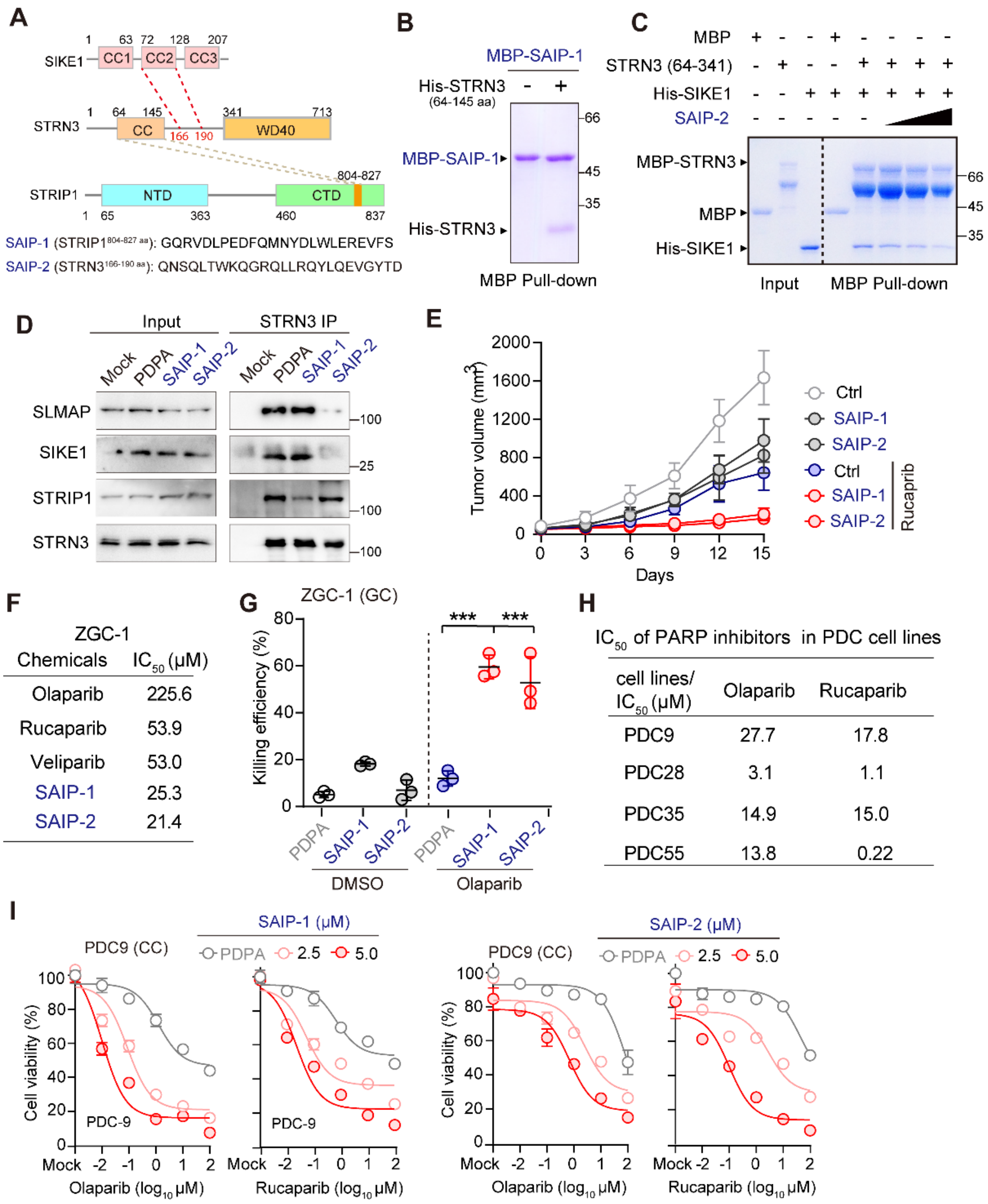

Figure S10. Co-targeting STRIPAK and PARP elicits synthetic lethality in human tumors. (**A**) A schematic illustration of the SAIP-1/2 peptides, derived from STRIP1 and STRN3, respectively. (**B**) In vitro pull-down assay showing the direct binding of SAIP-1 to STRN3. (**C**) In vitro MBP pull-down assay showing that SAIP-2 disrupted in a dose-dependent manner the SIKE1-STRN3 interaction. (**D**) STRN3 Co-IP assay showing that SAIP-1 and SAIP-2 disrupted the interactions of STRN3 with STRIP1 and SIKE1/SLMAP, respectively. (**E**) Curves of in vivo growth of tumors treated with SAIP-1/2 and rucaparib, either alone or combined. (**F**) IC_{50} values of SAIP-1/2 and PARPi in GC PDCs of the ZGC-1 cell line. (**G**) Killing efficiency plots resulting from ZGC-1 cells having been treated with SAIP-1/2 and olaparib, either alone or combined, for 24 h and then tested for cell viability. (**H**) IC_{50} values of PARPi in various colon cancer PDCs. (**I**) Cell viability plots for samples of PDC9 cells treated with various doses of, respectively, olaparib and rucaparib, each in the presence of, respectively, 0, 2.5, and 5.0 μM SAIP-1/2 for 24 h.

Related to **Figure 9**

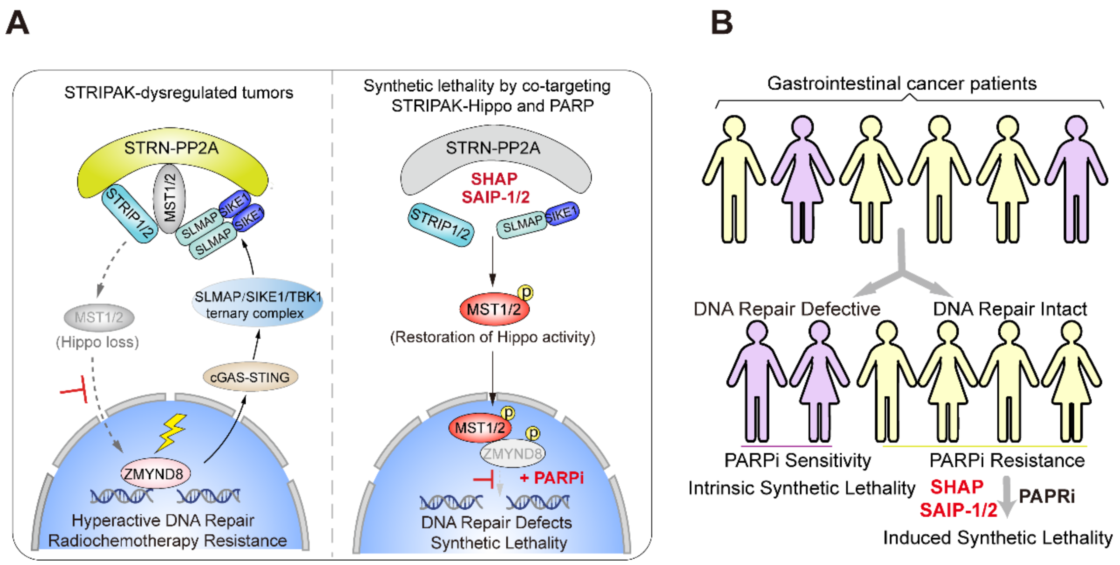

Figure S11. A working model for MST1/2-mediated restriction of DSB repair and antitumor strategy. (**A**) According to our model, in response to DNA damage, the cGAS-STING-TBK1 pathway relays a signal of damaged DNA to the STRIPAK-Hippo complex to facilitate a tightening of the complex via a TBK1-induced structural stabilization of the SIKE1-SLMAP arm. As such, STRIPAK-mediated dephosphorylation and inactivation of MST1/2 result in a ZMYND8-dependent DSB repair capacity and endows cancer cells with resistance to radio/chemotherapy (left panel). In contrast, targeting STRIPAK-MST1/2 assembly using peptide inhibitors such as SHAP or SAIP-1/2 releases the MST1/2 kinases so that they could translocate into the nucleus to suppress ZMYND8-dependent DSB repair, eliciting a synthetic lethality effect on the cancer cell when combined with PARP inhibitors (right panel). (**B**) Translational potency of simultaneous targeting of STRIPAK and PARP to induce synthetic lethality in human gastrointestinal tumors regardless of intrinsic DNA repair status. Such a combined strategy could be applied universally, i.e., to different types of tumors.

Supplementary Table 1. List of siRNA sequences used in the current work.

Gene name	siRNA sequence (5’-3’)
Control	UUCUCCGAACGUGUCACGU
MST1	GUAGUCAAAUAUUAUGGCATT
MST2	GCUGGUCAGUUAACAGAUATT
MST3	UGAAAGGACUCGAUUAUCU
MST4	GCCUGAUCCAAAGAAAGUATT
STK25	GCGCACUGCUGUUCAGAUA
MINK1	UGAAAUACGAGCGGAUUAA
TNIK	GAACAUACGGGCAAGUUUA
MAP4K4	AGAGCGACAGAGACATTTATT
PP2Aα	GAUCCCAGUAACAGGUCAU
PP2Ac	GGAACUUGACGAUACUCUATT
STRN	GCACAGAGGCUGAAGUUAATT
STRN3	GCCAGUUAACGUGGAAGCATT
STRN4	ACUCUAGCAUCUUGAUCCGTT
FGFR1OP2	GAGCAAGUACCGAGAACAAAU
CTTNBP2NL	ACCGTTGGTTGATCCCTACT
CTTNBP2	CCCGCAGGGCCTCGATGACA
SIKE1	CUUCGAGAAUUAUUGUCCATT
SLMAP	AUUUAGGUCUUGCUCAUCCTT
TRAF3IP3	GACCAACAAUUAAGAACGATT
STRIP1	CCAUGAAGAAAGUUCUCUUTT
STRIP2	GCAGCCGAGUUGUCAGAAUTT
cGAS	GCAAAAGUUAGGAAGCAAC
STING	UCAUAAACUUUGGAUGCUA
LATS1	GGUGAAGUCUGUCUAGCAA
LATS2	GCACGCAUUUUACGCCUUA
YAP	CUGCCACCAAGCUAGAUAATT
TAZ	ACGTTGACTTAGGAACTTT
ZMYND8-1	GGACUUUCCCCUUUUUAUA
ZMYND8-2	GAACAUAGAUGAAUGAAA
